# Cognitive Rehabilitation and Functional Outcomes in Long COVID–Related Cognitive Impairment: A Randomized Clinical Trial

**DOI:** 10.1001/jamanetworkopen.2026.20687

**Published:** 2026-07-01

**Authors:** Martina Vanova, Aysha Mohamed Rafik Patel, Iona Scott, Gina Gilpin, Emily N. Manning, Charlotte Ash, Philippa Wittenberg, Jason Lim, Zoe Hoare, Rachel Evans, Nathan Bray, Christopher M. Kipps, Ciara Devine, Saliha Ahmed, Ross Dunne, Anna Koniotes, Catherine Warren, Dennis Chan, Aida Suarez-Gonzalez

**Affiliations:** 1 Dementia Research Centre, University College London Queen Square Institute of Neurology, Faculty of Brain Sciences, University College London, London, United Kingdom; 2 School of Law, Social and Behavioural Sciences, Faculty of Business and Social Sciences, Kingston University, United Kingdom; 3 Institute of Cognitive Neuroscience, University College London, London, United Kingdom; 4 School of Applied Sciences, University of Brighton, United Kingdom; 5 North Wales Medical School, Bangor University, Bangor, United Kingdom; 6 School of Health Sciences, Bangor University, Bangor, United Kingdom; 7 Department of Neurology, University Hospitals Southampton National Health Service Trust, Southampton, United Kingdom; 8 Greater Manchester Dementia Research Centre, Greater Manchester Mental Health National Health Service Trust, Manchester, United Kingdom; 9 Geoffrey Jefferson Brain Research Centre, University of Manchester, Manchester, United Kingdom; 10 Department of Neurology, University Hospitals Sussex National Health Service Trust, United Kingdom

## Abstract

**Importance:**

Cognitive impairment is common in long COVID and severely affects daily life, with no proven treatments to date.

**Objective:**

To evaluate the ability of cognitive rehabilitation (CR) to improve goal attainment, cognitive, and clinical outcomes in individuals with cognitive impairment as part of long COVID.

**Design, Setting, and Participants:**

This multicenter, single-blind, 2-arm, parallel-group randomized clinical trial was conducted at 3 sites in England between February 2023 and March 2024. Participants were adults aged 30 to 60 years with prior COVID-19 infection and objective cognitive impairment (≥1 SD below age norm in ≥2 cognitive domains). A sample size of 88 participants (44:44) was required to detect a conservative effect of 0.7 on the goal attainment score at 3 months.

**Interventions:**

Participants were randomized (1:1) to CR or treatment as usual (TAU). CR consisted of 10 individual 1-hour sessions conducted once per week with a trained researcher, applying evidence-based strategies to 3 individually selected, personally meaningful functional goals. TAU was variable, with most participants having access to specialist memory clinics.

**Main Outcome and Measures:**

The primary outcome consisted of participant-reported goal-attainment scores at 3 months after randomization measured by the Bangor Goal-Setting Interview. Analysis was conducted on an intention-to-treat basis using multilevel mixed-effects models with 2-sided 95% CIs and 5% significance.

**Results:**

A total of 78 participants (24 male [30.8%] and 54 female [69.2%]; mean [SD] age, 47.3 [7.2] years) were randomized, including 38 individuals in CR and 40 individuals in TAU groups. At 3 months after randomization, goal attainment was significantly greater in the CR compared with the TAU group (adjusted mean difference, 2.88 [95% CI, 2.03-3.73]; *P* < .001; Cohen *d* = 1.57), with CR providing a large and clinically meaningful treatment effect. This was sustained at 6 months, with a lower effect size (adjusted mean difference, 1.72 [95% CI, 0.86-2.57]; *P* < .001; Cohen *d* = 0.91).

**Conclusions and relevance:**

In this study, individualized, goal-oriented CR led to significant and sustained improvements in goal attainment in people with long COVID–related cognitive impairment. These findings may guide and inform the provision of CR treatments and services for people living with long COVID.

**Trial Registration:**

ClinicalTrials.gov Identifier: NCT05731570

## Key Points

**Question:**

Does cognitive rehabilitation (CR) improve goal attainment, cognitive, and clinical outcomes in people with cognitive impairment as a part of long COVID?

**Findings:**

In this randomized clinical trial of 78 adults with prior COVID-19 infection and objective cognitive impairment, CR resulted in significantly greater functional goal attainment scores compared with treatment as usual at 3 months after randomization, and this significant difference remained at 6 months.

**Meaning:**

These findings suggest that CR can effectively treat cognitive impairment in long COVID.

## Teaser

This randomized clinical trial investigates the effect of cognitive rehabilitation on goal attainment, cognitive, and clinical outcomes in individuals with long COVID–related cognitive impairment.

## Introduction

Long COVID is defined as an infection-associated chronic condition that occurs after SARS-CoV-2 infection and is present for at least 3 months as a continuous, relapsing and remitting, or progressive disease that affects 1 or more organ systems.^1^ It can affect up to 36% of individuals with infections.^2^ Fatigue, myalgia, cognitive impairment (colloquially termed “brain fog”^3^), headache, anxiety, anosmia, and insomnia are among the most prominent symptoms.^4^ More than 27% of COVID-19 survivors develop persistent cognitive impairment,^5^ characterized by deficits in executive function, processing speed, attention, memory, visuospatial, and language abilities.^6^

The consequences of cognitive impairment in long COVID are profound at individual and societal levels and include reduced quality of life,^4^ loss of income, and work absenteeism.^7^ Effective treatments remain strikingly absent nearly 5 years from the onset of the COVID-19 pandemic, with most research consisting of case reports or observational studies.^8,9,10^ Of randomized clinical trials (RCTs) conducted, only hyperbaric oxygen therapy^11^ and meditation^12^ reported benefits in cognition, with small effect sizes and improvements observed only in the first few days after treatment. One study^13^ of a digital intervention observed secondary effects on processing speed but with no clear evidence of a meaningful treatment effect. No intervention has demonstrated sustained functional or cognitive gains.

Cognitive rehabilitation (CR) has been effective at treating memory, attention, and executive function in neurological conditions, such as stroke and multiple sclerosis.^14^ This supports CR use in long COVID, where those same deficits arise despite differing mechanisms (eg, inflammatory or vascular vs focal acute insult or demyelination mechanisms).^15^ However, to our knowledge, no study to date has evaluated the effectiveness of a structured, stand-alone CR program in this population. Best practice involves goal-oriented CR as an individualized, person-centered intervention that focuses on collaboratively identifying and targeting specific, meaningful functional goals relevant to the patient’s life. Progress is measured through goal attainment, providing a personalized indicator of functional change and treatment effectiveness.

This study aimed to evaluate the efficacy of individualized, goal-oriented CR for people with cognitive impairment associated with long COVID. We hypothesized that CR would improve self-reported goal attainment.

## Methods

### Trial Design

This was a multicenter, single-blind RCT with a 1:1 allocation ratio to CR or treatment as usual (TAU) conducted in 3 National Health Service (NHS) trusts in the UK between February 2023 and March 2024 under the name Cognitive Impairment in Long COVID: Phenotyping and Rehabilitation (CICERO). The trial was approved by the East of England–Essex Research Ethics Committee. The study protocol was published^16^ (see protocol and statistical analysis plan in Supplement 1) and prospectively registered. This study is reported following the Consolidated Standards of Reporting Trials (CONSORT) guideline for RCTs.

### Participants

Eligible participants were adults aged between 30 and 60 years with evidence of prior COVID-19 infection, self-reported cognitive symptoms persisting more than 3 months after infection,^1^ and scoring 1 or more SDs below age-adjusted norms in at least 2 cognitive domains.^16^ Exclusion criteria (factors affecting cognition) were presence of acute neurological disorder (eg, stroke or encephalitis), medication impacting cognition (ie, cholinesterase inhibitors, neuroleptics, and drugs with major sedative effects like opioids or benzodiazepines), preexisting major psychiatric or medical disorder, high alcohol intake (defined by the NHS and GOV.UK as >50 units/week for men or >35 units/week for women^17^), and recreational drug use. Written informed consent was obtained from all eligible participants before the cognitive domains assessment was carried out. Participants were recruited from research databases for the University Hospitals Sussex NHS Trust, Southampton General Hospital, Greater Manchester Mental Health NHS Foundation Trust, and National Institute for Health and Care Research and via social media. Ethnicity was self-reported as White and other ethnicity, which included Asian; Black, African, Caribbean, or Black British; multiple racial or ethnic groups; any other race or ethnicity; and prefer not to say. Due to small population sizes, races and ethnicities different from White were grouped into *other*. Collecting these data provides a baseline to monitor structural discrimination, ensure equal access to services, and hold institutions, such as the NHS and government bodies, accountable.

### Randomization and Masking

We used a dynamic adaptive algorithm and stratification per site and the trial overall. Randomization results were disclosed to the researcher conducting CR, who informed participants about their allocation. The statistical team (Z.H. and R.E.) and outcome measure raters (A.M.R.P., I.S., C.D., and S.A.) were blinded to participant group allocation.

### Procedures

At baseline, participants completed an online goal-setting interview using an adapted version of the Bangor Goal Setting Interview (BGSI)^18^ (eAppendix in Supplement 2) to set 3 personal goals. The BGSI is a manualized tool to identify Specific, Measurable, Achievable, Relevant, and Time-Bound (SMART) therapeutic goals. The BGSI goal-attainment scale rates each goal 1 to 10 points (low to high), with an improvement of 2 or more points considered clinically meaningful.^19^

### Cognitive Rehabilitation

Each participant received 10 weekly 1-hour telehealth sessions to work on 3 selected goals (eg, “I will write a report for 30 minutes without breaks, twice a week.”) often related to returning to work or improving work performance. Goals were addressed sequentially, with 3 sessions dedicated to each goal. The final session focused on reviewing progress, consolidating strategies, discussing gains maintenance after the intervention, and questions. CR strategies (eTable 5 in Supplement 2) were selected based on proven effectiveness in improving cognition in multiple sclerosis, stroke, and traumatic brain injury^14,20^ and refined in consultation with the patient-public involvement and engagement group (eTable 1 in Supplement 2). CR was delivered by a postdoctoral researcher (M.V.) trained for this study. Fidelity and the use of CR strategies were monitored through session logs and case discussions.

### Treatment as Usual

Participants in the control group received TAU, representing the standard of care provided by current clinical services for long COVID. TAU varied between individuals, reflecting differences in provision across health services, and ranged from pharmacological symptom management (eg, for headache and anxiety) to nonpharmacological treatment, such as physiotherapy and fatigue management. Other concomitant treatments are summarized in Table 1.

**Table 1.** Participant Characteristics and Outcome Measure Scores at Baseline

Variable	Participants, No. (%)		
	Overall (N = 78)	TAU (n = 40)	CR (n = 38)
**Participant characteristics**			
Age, mean (SD), y	47.3 (7.2)	47.5 (6.9)	47.1 (7.5)
Sex at birth			
Male	24 (30.8)	14 (35.0)	10 (26.3)
Female	54 (69.2)	26 (65.0)	28 (73.7)
Self-reported race and ethnicity			
White	67 (85.9)	34 (85.0)	33 (86.8)
Other^a^	11 (14.1)	6 (15.0)	5 (13.2)
Asian or Asian British	5 (6.4)	4 (10.0)	1 (2.6)
Black, African, Caribbean, or Black British	2 (2.6)	1 (2.5)	1 (2.6)
Multiple racial or ethnic groups	1 (1.3)	0	1 (2.6)
Any other race or ethnicity	1 (1.3)	0	1 (2.6)
Prefer not to say	2 (2.6)	1 (2.5)	1 (2.6)
Years of formal education, mean (SD)	16.6 (4.0)	16.7 (4.4)	16.5 (3.5)
Highest level of education			
University degree	41 (52.5)	21 (52.5)	20 (52.6)
Other (A-levels, GCSE, and others)	37 (47.5)	19 (47.5)	18 (47.4)
Disability^b^			
No disability	58 (74.4)	28 (70.0)	30 (79.0)
≥1 Disability	19 (24.4)	12 (30.0)	7 (18.4)
Occupation status			
Employed	62 (79.5)	28 (70.0)	34 (89.5)
Other (unemployed, retired, or prefer not to say)	16 (20.5)	12 (30.0)	4 (10.5)
Drugs^b^			
No drugs	40 (51.3)	25 (62.5)	15 (39.5)
≥1 Drug taken	37 (47.4)	14 (35.0)	23 (60.5)
Drug type^c^^,^^d^			
Drugs for mental health	37 (47.4)	14 (35.0)	23 (60.5)
Antiepileptics	2 (2.6)	0 (0)	2 (5.3)
Other	40 (51.3)	25 (62.5)	15 (39.5)
Time (based on first symptom) since acute infection, mean (SD), mo	28.0 (7.7)	27.3 (8.6)	28.7 (6.7)
COVID-19 respiratory symptoms^c^			
None	11 (14.1)	5 (12.5)	6 (15.8)
Respiratory symptoms, no home assistance	48 (61.5)	23 (57.5)	25 (65.8)
Respiratory symptoms, with home assistance	13 (16.8)	8 (20.0)	5 (13.2)
Hospitalized but not put on a ventilator	5 (6.4)	4 (10.0)	1 (2.6)
Depression criteria at baseline (PHQ-8)			
Minimal or no depression (0-4)	6 (7.7)	3 (7.5)	3 (7.9)
Mild depression (5-9)	15 (19.2)	8 (20.0)	7 (18.4)
Moderate depression (10-14)	22 (28.2)	10 (25.0)	12 (31.6)
Moderately severe depression (15-19)	25 (32.1)	15 (37.5)	10 (26.3)
Severe depression (20-24)	10 (12.8)	4 (10.0)	6 (15.8)
Concomitant treatment^e^			
Fatigue management training	73 (93.6)	38 (95)	35 (92.1)
Cognitive behavioral therapy	6 (7.7)	3 (7.5)	3 (7.9)
Talking therapy	4 (5.1)	1 (2.5)	3 (7.9)
Positive hypnotherapy	1 (1.3)	0	1 (2.6)
**Outcome measure scores, mean (SD)**			
BGSI			
Goal attainment	2.9 (1.0)	2.9 (1.1)	2.9 (0.9)
Goal satisfaction, median (IQR)^f^	2.3 (1.7-2.7)	2.3 (1.7-2.7)	2.3 (1.7-2.7)
Goal readiness, median (IQR)^f^	9.3 (8.0-10.0)	9.3 (7.7-10.0)	9.5 (8.7-10.0)
Goal difficulty, median (IQR)^f^	6.8 (1.3)	6.9 (1.4)	6.7 (1.2)
Goal importance, median (IQR)^f^	9.3 (8.3-10.0)	9.2 (8.3-9.8)	9.3 (8.7-10.0)
LSA	5.1 (1.8)	5.0 (1.3)	5.1 (1.3)
SF-DEM	21.1 (5.9)	20.9 (5.9)	21.7 (6.0)
IADL, median (IQR)^f^	6.5 (6.0-8.0)	6.0 (5.0-7.0)	7.0 (6.0-8.0)
GAD-7	9.3 (6.3)	8.8 (5.8)	9.7 (6.9)
PHQ-8	13.1 (5.6)	13.2 (5.4)	13.1 (5.8)
CFS, median (IQR)^f^	36.0 (32.0-40.0)	36.0 (31.5-40.0)	35.5 (32.0-39.0)
PSQI	10.6 (4.2)	10.3 (4.6)	10.9 (3.7)
RBANS total index score, median (IQR)^f^	94.5 (83.0-102.5)	93.0 (78.0-101.0)	95.0 (86.0-104.0)
D-KEFS			
Trail making			
Condition 2, number sequencing	7.6 (3.2)	8.1 (3.2)	7.2 (3.2)
Condition 4, number-letter sequencing	8.1 (3.3)	8.0 (3.1)	8.2 (3.5)
Color word			
Condition 1, color naming, median (IQR)^f^	6.0 (3.0-9.0)	6.5 (4.0-9.0)	6.0 (2.0-8.0)
Condition 2, word reading	6.7 (3.6)	6.8 (3.6)	6.5 (3.7)
Condition 3, inhibition	7.2 (4.2)	7.6 (4.2)	6.8 (4.2)
Condition 4, inhibition switching	7.0 (3.9)	7.1 (4.1)	7.0 (3.6)
Verbal fluency			
Condition 1, letter fluency	9.6 (3.7)	9.2 (4.1)	10.0 (3.3)
Condition 2, category fluency	10.7 (3.9)	10.5 (3.7)	10.8 (4.0)
Condition 3, category switching (total correct responses)	10.5 (3.3)	10.6 (3.1)	10.4 (3.5)
Condition 3_ta, category switching (total accuracy)	10.8 (2.9)	11.1 (2.7)	10.6 (3.1)
WAIS	16.1 (3.9)	15.9 (3.6)	16.2 (4.3)
DSQ-PEM (PEM)			
Present	4 (5.1)	3 (7.5)	1 (2.6)
Not present	74 (94.9)	37 (92.5)	37 (97.4)
DSQ-PEM (ME/CFS)			
Not present	34 (43.6)	19 (47.5)	15 (39.5)
Present	44 (56.4)	21 (52.5)	23 (60.5)
4 Mountains Test	8.2 (2.49)	7.7 (2.10)	8.8 (2.76)
**Gorilla tasks, percentile**			
Task 1 (simple RT)			
≤Fifth	43 (55.1)	20 (50.0)	23 (60.5)
Sixth to 100th	31 (39.7)	17 (42.5)	14 (36.8)
Missing	4 (5.1)	3 (7.5)	1 (2.6)
Task 2 (inhibition)			
≤Fifth	37 (47.4)	15 (37.5)	22 (57.9)
Sixth to 100th	37 (47.4)	22 (55.0)	15 (39.5)
Missing	4 (5.1)	3 (7.5)	1 (2.6)
Task 3 (XNA), condition 1			
≤Fifth	17 (21.8)	8 (20.0)	9 (23.7)
Sixth to 100th	57 (73.1)	29 (72.5)	28 (73.7)
Missing	4 (5.1)	3 (7.5)	1 (2.6)
Task 3 (XNA), condition 2			
≤Fifth	33 (42.3)	16 (40.0)	17 (44.7)
Sixth to 100th	41 (52.6)	21 (52.5)	20 (52.6)
Missing	4 (5.1)	3 (7.5)	1 (2.6)

Abbreviations: BGSI, Bangor Goal-Setting Interview; CFS, Chronic Fatigue Scale; CR, cognitive rehabilitation; D-KEFS, Delis-Kaplan Executive Function System; DSQ-PEM, DePaul Symptom Questionnaire, Post-Exertional Malaise; GAD-7, Generalized Anxiety Disorder-7; GCSE, General Certificate of Secondary Education; IADL, Instrumental Activities of Daily Living; LSA, Life Space Assessment; ME/CFS, myalgic encephalomyelitis and chronic fatigue syndrome; PHQ-8, Patient Health Questionnaire-8; PSQI, Pittsburgh Sleep Quality Index; RBANS, Repeatable Battery for the Assessment of Neuropsychological Status; RT, reaction time; SF-DEM, Social Functioning in Dementia Scale; TAU, treatment as usual; WAIS, Wechsler Adult Intelligence Scale; XNA, working memory task.

^a^ Other race and ethnicity included Asian or Asian British; Black, African, Caribbean, or Black British; multiple racial or ethnic groups; any other race or ethnicity; and prefer not to say.

^b^ Because of missing data, values do not account for 100% (eTable 3 in Supplement 1).

^c^ Participants could select several options.

^d^ Drug types included cholinesterase inhibitors, drugs for mental health (antidepressants, anxiolytics, and other mood stabilizers except neuroleptics), antiepileptics, and other. Cholinesterase inhibitors were combined with other due to low numbers.

^e^ Therapy: cognitive-behavioral therapy, talking therapy, or positive hypnotherapy.

^f^ Because data are not normally distributed, the median and IQR are given. For normally distributed data, the mean (SD) is reported.

### Outcome Measures

#### Primary and Secondary Outcome Measures

The primary effectiveness outcome was participant-reported goal attainment of 3 goals assessed at 3 months and 6 months after randomization to determine any sustained treatment effect. Secondary outcomes were measured at baseline and 3 and 6 months after randomization and comprised the following measures:

#### Cognitive Measures

This included established cognitive tests and novel assessments. The Repeatable Battery for the Assessment of Neuropsychological Status (RBANS)^21^ tested immediate memory, visuospatial memory, language, attention, and episodic memory. The Delis–Kaplan Executive Functioning System (D-KEFS)^22^ incorporated the Trail Making Test of Cognitive Flexibility, Color-Word Interference Test of Inhibitory Control and Cognitive Flexibility, and Verbal Fluency Test of Verbal and Semantic Processing. The Wechsler Adult Intelligence Scale-III^23^ Digit Span assessed working memory and attention, and the Test of Premorbid Functioning^24^ was used for premorbid IQ. Novel cognitive testing included the Long COVID Cognitive Assessment Battery,^25^ a short battery evaluating cognitive speed, inhibitory control, attention, and working memory; it was designed to accommodate the fatigue effect and was previously tested in long COVID (90% sensitivity), administered using the Gorilla platform.^26^ The 4 Mountains Test,^27^ a novel test of allocentric spatial memory, was also administered to test hippocampal function; it was previously tested on Alzheimer disease (100% sensitivity and 78% specificity).

#### Clinical and Functional Measures

We used Quality of Life (EQ-5D-5L)^28^ for health quality, the Life Space Assessment^29^ for functional mobility, and Social Functioning^30^ for social activity. The Instrumental Activities of Daily Living Scale^31^ assessed functional independence, while the Generalized Anxiety Disorder Assessment^32^ measured anxiety symptom and the Patient Health Questionnaire^33^ assessed depressive symptoms. The Chalder Fatigue Scale^34^ evaluated mental and physical fatigue, while the Pittsburgh Sleep Quality index^35^ assessed sleep disturbance and the DePaul Symptom Questionnaire–Post-Exertional Malaise assessed symptoms of mental and cognitive exhaustion.^36^

#### Measure of Service Use

The Client Service Receipt Inventory^37^ collected information about health care services used for a future health-economic analysis. Goal satisfaction, readiness to work on each goal, and perceived difficulty of goals (all scored on a scale of 1-10 points) were also analyzed and used as covariates in models. Intervention adherence measures included session attendance, strategies practiced in each session, and home practice logs that were self-reported between sessions.

#### Sample Size

Based on a 2-sample *t* test assuming equal variance in Power Analysis and Sample Size (PASS) version 15.0.10 (Number Cruncher Statistical Systems), a sample size of 88 participants (44:44) was required to detect a conservative effect of 0.7 on the goal attainment score at 3 months, with 90% power at 5% significance. In the absence of prior studies assessing CR in long COVID, this power calculation used an effect size based on the Goal-Oriented Cognitive Rehabilitation in Early-Stage Alzheimer’s and Related Dementias: Multicentre Single-Blind Randomised Controlled Trial (GREAT)^19^ (Cohen *d* = 0.8) for goal attainment after CR in Alzheimer disease. Analyses followed the intention-to-treat principle.

#### Missing Data

Multiple imputation for missing data at baseline and 3 and 6 months was used for self-report outcomes. Analysis model covariates (group, site, age, education level, and baseline RBANS score) and identified predictors of missing data defined in the statistical analysis plan (Supplement 1) were included in imputation models.^11^

Missing data were handled via multiple imputation by chained equations (MICE) in Stata statistical software version NNN (StataCorp), incorporating all 3 times (baseline, 3 months, and 6 months). The number of imputations matched the maximum percentage of missing data across times.^38^ To assess the robustness of missing-at-random assumptions, complete-case sensitivity analyses were performed for the primary outcome and significant secondary outcomes.

### Statistical Analysis

Statistical analysis took place between November 2024 and May 2025. Primary analysis was conducted on the intention-to-treat population using multiple imputation techniques.

For all outcomes, continuous variables were analyzed using multilevel (mixed-effect) linear regression models, and categorical (binary) measures were analyzed using multilevel (mixed) logistic regression models. Models included stratification variable (site) as a random effect, along with sex, age (continuous), years of education (continuous), and RBANS composite score at baseline as fixed factors. Due to low recruitment at 2 locations, the site was collapsed into a binary variable: lead site (Sussex) vs other site (Manchester or Southampton). Participant identities were not included as random effects, but baseline scores of outcomes were incorporated in models to account for baseline differences. Furthermore, when analyzing reaction times, it was important to account for error rates (ie, the number of errors participants made). Therefore, baseline and follow-up error counts were included where applicable.

In line with the prespecified statistical analysis plan (Supplement 1), we did not correct for multiple comparisons given that only 1 primary outcome was defined a priori. Analyses of secondary outcomes were exploratory, and any results related to these outcomes should be interpreted with caution. For all analyses, 2-sided 95% CIs with 5% significance were used.

We conducted 3 sensitivity analyses: (1) reanalysis of the primary and significant secondary outcomes using complete case data, (2) restriction to participants assessed within 14 days before or after the planned follow-up, and (3) exclusion of participants who did not receive all 10 intervention sessions within a 12-week window. Exploratory analyses examined associations of readiness, importance, and difficulty (mean across 3 goals at baseline) with goal attainment. Clinical response was explored through descriptive subgroup analyses. Participants improving by 2 or more points were classified as *responders*, and those improving by 4 or more points were classified as *major responders*.

## Results

### Participants

**Figure 1.**
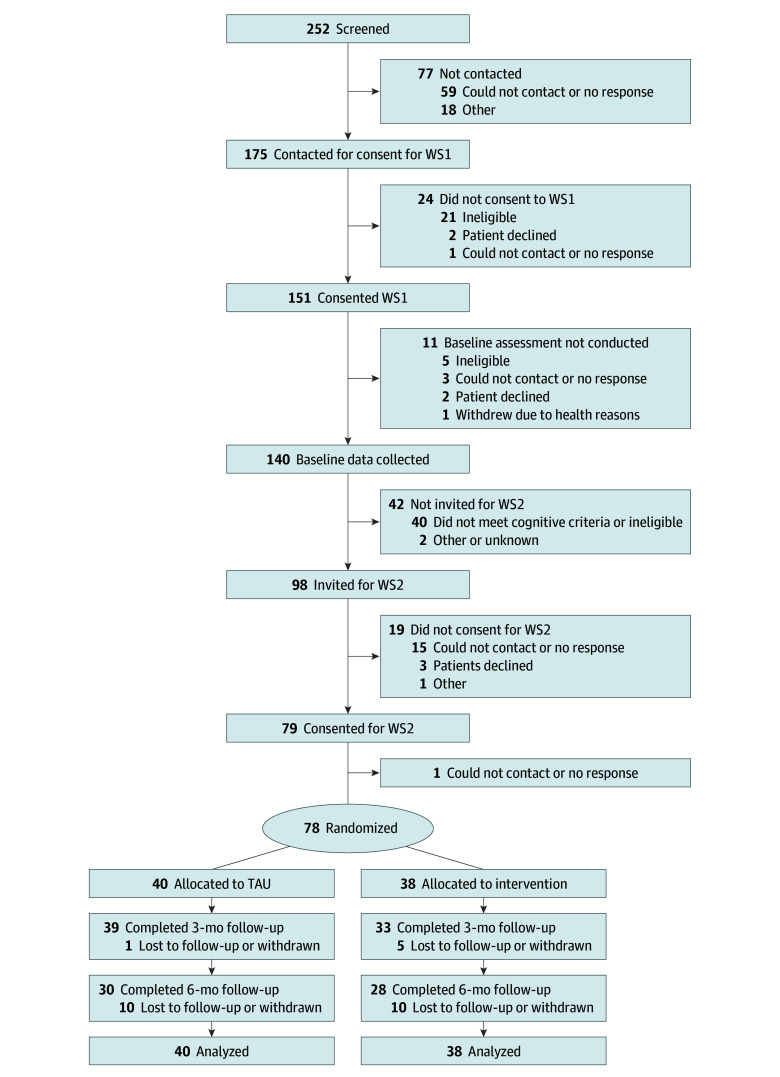
Study Flowchart TAU indicates treatment as usual; WS, work stream.

Among 258 participants screened for eligibility, 78 individuals (mean [SD] age, 47.3 [7.2] years; 24 male [30.8%] and 54 female [69.2%]; 67 White [85.9%] and 11 other ethnicity [14.1%]) were randomized (Figure 1) and included in intention-to-treat analysis; there were 40 participants in TAU and 38 in CR groups. At 3 months, completion rates were 39 participants (97.5%) in TAU and 33 participants in CR (86.8%) groups (Figure 1). By 6 months, these rates declined to 30 participants (75.0%) in TAU and 28 participants (73.7%) in CR groups. Participants had a mean (SD) 16.6 (4.0) years of education, and 77 participants (98.7%) spoke English as their first language. Full demographic information is shown in Table 1.

### Descriptives: Baseline Data

At baseline, CR and TAU groups had low mean (SD) goal attainment scores (CR: 2.9 [0.9]; TAU:2.9 [1.1]) and median (IQR) goal satisfaction scores (2.2 [1.7-2.7] for both groups). All baseline scores are descriptively reported in Table 1. Most of the CR group (32 participants) received all 10 intervention sessions, while 4 received 1 to 4 sessions and 2 withdrew from the CR.

### Data Processing

Data met assumptions for the MICE model and were deemed to be missing at random. For 6-month cognitive measures, missing data exceeded 40%,^11^ multiple imputation was not used, and complete case analysis is presented (eTable 2 in Supplement 2). The level of missing data at baseline and follow-ups overall and by group is presented in eTable 3 in Supplement 2.

All assumptions for underlying statistical models were checked and held. Robust standard errors (SEs) were used. For categorical measures, the assumption of proportional odds held for most models; for violations, the outcome was recoded into a binary variable (Gorilla task, condition 2). For categorical outcomes, models were run incorporating site as a fixed factor.

### Primary Outcome

At 3 months, adjusted mean (SE) goal attainment scores were significantly higher in the CR (7.84 [0.19]) than the TAU (4.97 [0.53]) group, with an adjusted mean difference of 2.88 (95% CI, 2.03-3.73; *P* < .001) and a large treatment effect size (Cohen *d* = 1.57). At 6 months, the adjusted mean difference remained significant (1.72 [95% CI, 0.86-2.57]; *P* < .001) and treatment effect remained large (Cohen *d* = 0.91), with improvement in the TAU group (adjusted mean [SE], 5.74 [0.31]), while the CR group remained stable (adjusted mean [SE], 7.46 [0.36]).

### Secondary Outcomes

The CR group reported higher goal satisfaction scores than the TAU group at 3 months (adjusted mean difference, 2.87 [95% CI, 2.08-3.65]; *P* < .001; Cohen *d* = 1.48) and 6 months (adjusted mean difference, 1.53 [95% CI, 0.56-2.50]; *P* < .01; Cohen *d* = 0.72). For cognitive outcomes, the CR group scored higher than the TAU group on executive function, as measured by the D-KEFS Trail Making C4 Number-Letter Sequencing, at 3 months (adjusted mean difference, 0.87 [95% CI, 0.21-1.52]; *P* = .01; Cohen *d* = 0.48) and 6 months (adjusted mean difference, 0.93 [95% CI, 0.75-1.11]; *P* = .002; Cohen *d* = 0.18). At 6 months, the CR group also showed higher processing speed on condition 2 (number sequencing; adjusted mean difference, 1.90 [95% CI, 0.32-3.49]; *P* = .02; Cohen *d* = 0.07). There were no significant group differences on other cognitive tests or symptoms, including fatigue, postexertional malaise, anxiety, depression, and sleep disturbance at 3 or 6 months (Table 2).

**Table 2.** Clinical Effectiveness Outcomes^a^

Outcome, dataset, and time	Participants, No.	Maximum likelihood estimate		Adjusted value, mean (SE)		Absolute Cohen *d*
		Coefficient (SE) [95% CI]	*P* value	TAU	CR	
Goal attainment (BGSI)						
MI at 3 mo	78	2.88 (0.43) [2.03 to 3.73]	<.001	4.97 (0.53)	7.84 (0.19)	1.57
MI at 6 mo	78	1.72 (0.43) [0.86 to 2.57]	<.001	5.74 (0.31)	7.46 (0.36)	0.91
Goal satisfaction (BGSI)						
MI at 3 mo	78	2.87 (0.4) [2.08 to 3.65]	<.001	4.59 (0.27)	7.46 (0.30)	1.48
MI at 6 mo	78	1.53 (0.49) [0.56 to 2.50]	.002	5.61 (0.33)	7.14 (0.36)	0.72
LSA						
MI at 3 mo	78	−0.36 (0.36) [−1.06 to 0.34]	.31	4.88 (0.40)	4.51 (0.43)	0.12
MI at 6 mo	78	−0.69 (0.38) [−1.43 to 0.06]	.07	5.63 (0.25)	4.95 (0.30)	0.53
SF-DEM						
MI at 3 mo	78	1.81 (1.12) [−0.38 to 4.00]	.11	20.74 (0.74)	22.55 (0.81)	0.41
MI at 6 mo	78	−0.42 (1.21) [−2.79 to 1.96]	.73	22.45 (1.07)	22.03 (1.33)	0.13
IADL Scale						
MI at 3 mo	78	−0.35 (0.27) [−0.89 to 0.19]	.20	6.74 (0.12)	6.40 (0.18)	0.18
MI at 6 mo	78	−0.34 (0.3) [−0.92 to 0.24]	.25	6.63 (0.22)	6.30 (0.20)	0.03
GAD-7^b^						
MI at 3 mo	78	−0.89 (0.95) [−2.74 to 0.97]	.35	9.10 (1.04)	8.21 (0.63)	0.32
MI at 6 mo	78	−0.11 (0.95) [−1.99 to 1.77]	.91	8.14 (0.74)	8.03 (0.72)	0.02
PHQ-8^b^						
MI at 3 mo	78	0.18 (0.87) [−1.53 to 1.89]	.83	12.32 (1.03)	12.50 (1.11)	0.17
MI at 6 mo	78	0.89 (1.04) [−1.15 to 2.94]	.39	11.31 (1.13)	12.20 (0.79)	0.13
CFS^b^						
MI at 3 mo	78	−1.55 (1.78) [−5.03 to 1.94]	.38	32.47 (1.23)	30.93 (1.32)	0.34
MI at 6 mo	78	1.29 (1.7) [−2.08 to 4.65]	.45	28.99 (1.06)	30.27 (1.19)	0.07
PSQI^b^						
MI at 3 mo	78	−1.16 (0.7) [−2.54 to 0.22]	.10	10.79 (0.47)	9.63 (0.52)	0.23
MI at 6 mo	78	0.13 (0.83) [−1.51 to 1.77]	.88	10.09 (0.62)	10.22 (0.58)	0.25
DSQ-PEM, presence of PEM (yes or no), OR (SE) [95% CI]^c^						
MI at 3 mo	78	0.85 (0.65) [0.19 to 3.85]	.83	NA	NA	0.08
MI at 6 mo	78	3.27 (2.82) [0.60 to 17.87]	.17	NA	NA	0.37
RBANS total index score						
MI at 3 mo	78	0.19 (2.08) [−3.90 to 4.27]	.93	99.15 (1.75)	99.34 (1.98)	0.28
CC at 6 mo	43	−0.53 (2.82) [−6.05 to 5.00]	.85	105.99 (2.62)	105.46 (2.76)	0.39
D-KEFS						
Trail making						
Condition 2, number sequencing						
MI at 3 mo	78	0.41 (0.56) [−0.68 to 1.51]	.46	8.41 (0.50)	8.82 (0.56)	0.05
CC at 6 mo	43	1.9 (0.81) [0.32 to 3.49]	.02	9.07 (0.50)	10.97 (0.58)	0.07
Condition 4, number-letter sequencing						
MI at 3 mo	78	0.87 (0.33) [0.21 to 1.52]	.01	9.05 (0.35)	9.91 (0.34)	0.48
CC at 6 mo	43	0.93 (0.09) [0.75 to 1.11]	<.001	10.45 (0.38)	11.38 (0.48)	0.18
Color word						
Condition 1, color naming						
MI at 3 mo	78	−0.09 (0.66) [−1.39 to 1.21]	.89	7.56 (0.39)	7.47 (0.53)	0.14
CC at 6 mo	40	1.14 (0.71) [−0.25 to 2.53]	.11	8.90 (0.95)	10.04 (0.99)	0.14
Condition 2. word reading						
MI at 3 mo	78	−0.50 (0.44) [−1.40 to 0.39]	.26	7.56 (0.27)	7.05 (0.34)	0.07
CC at 6 mo	40	0.17 (0.13) [−0.08 to 0.41]	.18	9.06 (1.44)	9.23 (1.56)	0.16
Condition 3, inhibition						
MI at 3 mo	78	0.34 (0.92) [−1.47 to 2.14]	.72	8.50 (0.46)	8.84 (0.52)	0.01
CC at 6 mo	40	−0.41 (0.61) [−1.60 to 0.79]	.51	10.98 (1.12)	10.58 (1.73)	0.27
Condition 4, inhibition switching						
MI at 3 mo	78	0.83 (0.7) [−0.56 to 2.22]	.24	8.55 (0.29)	9.37 (0.74)	0.14
CC at 6 mo	40	0.85 (0.69) [−0.50 to 2.19]	.22	9.59 (0.42)	10.44 (0.50)	0.08
Verbal fluency						
Condition 1, letter fluency						
MI at 3 mo	78	−0.87 (0.86) [−2.56 to 0.81]	.31	11.47 (0.43)	10.59 (1.21)	0.03
CC at 6 mo	43	−1.31 (0.73) [−2.73 to 0.12]	.07	12.65 (1.24)	11.34 (1.25)	0.04
Condition 2, category fluency						
MI at 3 mo	78	0.12 (0.65) [−1.17 to 1.40]	.86	11.10 (0.42)	11.21 (0.51)	0.10
CC at 6 mo	43	−0.17 (0.22) [−0.59 to 0.26]	.44	12.54 (1.18)	12.37 (0.96)	0.06
Condition 3_tc, category switching (total correct responses)						
MI at 3 mo	78	−0.57 (0.48) [−1.55 to 0.42]	.25	10.92 (0.34)	10.35 (0.54)	0.14
CC at 6 mo	43	−1.54 (0.95) [−3.4 to 0.33]	.11	13.01 (1.12)	11.47 (1.16)	0.37
Condition 3_ta category switching (total accuracy)						
MI at 3 mo	78	−0.63 (0.46) [−1.56 to 0.30]	.18	11.13 (0.20)	10.49 (0.41)	0.21
CC at 6 mo	43	−1.78 (0.97) [−3.68 to 0.12]	.07	12.55 (0.61)	10.78 (0.70)	0.55
WAIS						
MI at 3 mo	78	0.36 (0.52) [−0.65 to 1.38]	.48	16.18 (0.34)	16.54 (0.36)	0.14
CC at 6 mo	43	−0.49 (0.78) [−2.01 to 1.04]	.53	17.17 (0.49)	16.68 (0.56)	0.04
4 Mountains Test^b^						
MI at 3 mo	78	−0.22 (0.38) [−0.99 to 0.54]	.56	7.06 (0.61)	6.83 (0.49)	0.25
CC at 6 mo	44	0.36 (0.64) [−0.89 to 1.61]	.57	6.61 (0.08)	6.98 (0.55)	0.66
Gorilla tasks, OR (SE) [95% CI]						
Task 1 (simple reaction time)^b^^,^^d^						
MI at 3 mo	78	0.84 (0.46) [0.29 to 2.45]	.75	NA	NA	0.03
CC at 6 mo	42	0.38 (0.25) [0.11 to 1.36]	.14	NA	NA	0.30
Task 2 (inhibition)^b^^,^^d^						
MI at 3 mo	78	0.87 (0.42) [0.34 to 2.27]	.78	NA	NA	0.03
CC at 6 mo	42	0.4 (0.27) [0.11 to 1.46]	.17	NA	NA	0.38
Task 3 (XNA), condition 1^b^^,^^d^						
MI at 3 mo	78	1.39 (0.63) [0.57 to 3.39]	.47	NA	NA	0.39
CC at 6 mo	42	0.91 (0.56) [0.27 to 3.07]	.88	NA	NA	0.12
Task 3 (XNA), condition 2^b^^,^^d^						
MI at 3 mo	78	1.69 (1.21) [0.41 to 6.95]	.46	NA	NA	0.46
CC at 6 mo	42	0.48 (0.3) [0.14 to 1.66]	.25	NA	NA	0.21

Abbreviations: BGSI, Bangor Goal-Setting Interview; CC, complete case; CFS, Chronic Fatigue Scale; CR, cognitive rehabilitation; D-KEFS, Delis-Kaplan Executive Functioning System; DSQ-PEM, DePaul Symptom Questionnaire, Post-Exertional Malaise; GAD-7, Generalized Anxiety Disorder-7; IADL, Instrumental Activities of Daily Living; LSA, Life Space Assessment; MI, multiple imputation; NA, not applicable; OR, odds ratio; PHQ-8, Patient Health Questionnaire-8; PSQI, Pittsburgh Sleep Quality Index; RBANS, Repeatable Battery for the Assessment of Neuropsychological Status; SE, standard error; SF-DEM, Social Functioning in Dementia Scale; TAU, treatment as usual (standard care); WAIS, Wechsler Adult Intelligence Scale; XNA, working memory task.

^a^ Results of primary regression models. The analysis model is a mixed-effect regression model.

^b^ Higher scores indicate worse outcome (ie, higher anxiety or depression severity, higher extent of fatigue, severe difficulties, or longer reaction times).

^c^ The model is logistic regression (with OR reported).

^d^ The model is ordinal logistic regression (with OR reported).

### Sensitivity Analyses

Sensitivity analysis 1 evaluated the impact of multiple imputation on the primary outcome and significant secondary outcomes (goal attainment, goal satisfaction, and D-KEFS executive function tests). For goal attainment, adjusted mean differences favored the CR group at 3 months (2.73 [95% CI, 1.66-3.79]; *P* < .001) and 6 months (1.70 [95% CI, 0.81-2.58]; *P* < .001). Goal satisfaction results were also consistent at 3 months (adjusted mean difference, 2.74 [95% CI, 1.53-3.95]; *P* < .001) and 6 months (adjusted mean difference, 1.40 [95% CI, 0.77-2.04]; *P* < .001). The only secondary cognitive outcome that remained significant at 3 months was D-KEFS Trail Making C4 Number-Letter Sequencing (adjusted mean difference, 1.22 [95% CI, 0.65-1.80]; *P* < .001), favoring the CR group. Effect sizes were comparable, with slight variation in estimates (eTable 2 in Supplement 2).

Sensitivity analysis 2 (eTable 2 in Supplement 2) included 27 participants with data collected within the prespecified 3-month time frame and 33 participants at 6 months. Goal attainment remained significantly higher in the CR group at 3 months (adjusted mean difference, 3.40 [95% CI, 3.14-3.65]; *P* < .001) and 6 months (adjusted mean difference, 1.29 [95% CI, 0.14-2.43]; *P* = .03). Sensitivity analysis 3 (eTable 2 in Supplement 2) included only 24 participants who completed the 12-week intervention within the prespecified time frame, giving a total sample of 62 participants (24 in the CR group and 38 in the TAU group). Goal attainment at 3 months remained significantly higher in the CR group (adjusted mean difference, 2.88 [95% CI, 2.28-3.47]; *P* < .001).

### Exploratory Analyses and Harms

In exploring factors associated with goal attainment, we found that increased baseline readiness and perceived difficulty were associated with greater goal attainment at 3 months (eTable 4 in Supplement 2) but not at 6 months. Importance was not associated with goal attainment at either time. The main effect of the intervention at 3 months remained significant after adjusting for these variables (adjusted mean difference, 2.62 [95% CI, 1.70-3.55]; *P* < .001).

**Figure 2.**
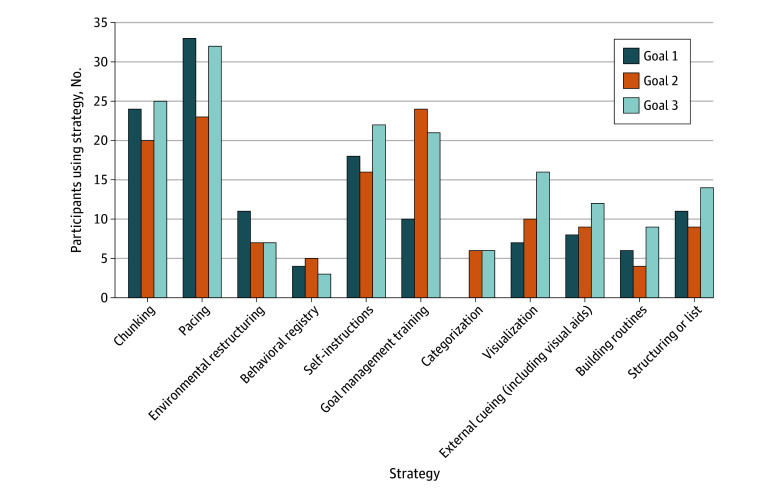
Cognitive Rehabilitation Strategies Used During Intervention Sessions Bars indicate the number of participants who used each strategy at least once for each of 3 therapy goals.

In exploring clinically meaningful change in goal attainment, we found that 32 participants in the CR group (84.2%) improved by 2 or more points in goal attainment scores at 3 months after baseline (ie, were responders) compared with 21 participants in the TAU group (52.5%). By 6 months, this proportion fell to 27 participants (71.0%) in the CR group and increased to 23 participants (57.5%) in the TAU group. Using a more stringent threshold of 4 or more points in improvement, 25 participants in the CR group (65.8%) met this criterion at 3 months compared with 6 participants in the TAU group (15.0%). At 6 months, 20 participants in the CR group (52.6%) met this criterion compared with 6 participants in the TAU group (15.0%). No inferential statistics were conducted given that these analyses were exploratory. Cognitive rehabilitation strategies used in the CR are summarized in Figure 2. Participants reported no trial-related adverse events.

## Discussion

In this RCT, goal-oriented cognitive rehabilitation improved self-reported goal attainment in people with long COVID and cognitive impairment, with a large effect at 3 months (Cohen *d* = 1.57) vs TAU, sustained at 6 months (Cohen *d* = 0.91). This demonstrates a clinically meaningful and durable benefit. Crucially, these findings suggest that targeting individually selected functional goals ensures clinical relevance. These findings are clinically important given the high prevalence of cognitive impairment in individuals with long COVID and its impact on quality of life and everyday functioning.^4^ Participants receiving CR reported greater satisfaction with their goal performance, reflecting the value of personalized outcomes. In chronic disorders, satisfaction can strengthen engagement with therapy, motivation, and long-term adjustment, and relate more closely to quality of life than performance alone.^6^

CR produced a small improvement in cognitive flexibility at 3 months (Cohen *d* = 0.48) and processing speed at 6 months (Cohen *d* = 0.07) of uncertain clinical relevance given near-normal baseline performance. There was no benefit in other cognitive domains (memory, language, attention, or verbal fluency) or symptoms (fatigue, sleep disturbance, anxiety, or depression). Gains in goal attainment likely reflect the acquisition of task-specific strategies and intervention-related psychological factors, suggesting more efficient use of limited cognitive resources rather than increased capacity.

Participants in the TAU group also showed a clinically meaningful improvement in goal attainment at 3 months despite no active intervention. While this may partly reflect the natural history of long COVID, it may also reflect the effect of identifying meaningful goals, which may have prompted self-initiated strategies.

### Positive Goal-Specific Outcomes

This study differs from previous work in 3 ways. First, we recruited individuals with cognitive impairment given that this is a major contributor to reduced daily function,^3,6^ which was the main reason for referral to specialist long COVID services. Second, we used goal-oriented cognitive rehabilitation, with participant-defined outcomes. Third, remote telehealth delivery of the intervention proved feasible and provided an opportunity to improve access to treatment. However, future implementation considerations will need to address the risk of digital exclusion and potential associated health inequalities.

### Limitations

We acknowledge some limitations typical of rehabilitation trials. First, participants could not be blinded to group allocation, which is common in behavioral interventions and may introduce expectancy effects. However, meaningful improvement in both groups and a large and sustained between-group difference make this expectancy effect unlikely to fully explain the findings. Second, outcome raters were intended to remain blinded, but this was sometimes compromised. Notably, raters were not involved in treatment delivery, reducing the risk of bias related to therapist-participant contact (eg, social desirability). Third, the absence of an active control condition meant greater therapist contact in the intervention group, which may have introduced nonspecific effects. However, the structured, strategy-based nature of CR and the magnitude and persistence of effects make this potential bias unlikely to account for reported findings. Fourth, although we aimed to recruit a diverse sample, participants were predominantly White British and university educated, limiting generalizability.

## Conclusions

In this RCT, CR improved self-reported goal-specific functional outcomes in long COVID. These effects were clinically meaningful and sustained at 6 months. Together, these findings provide the first class II evidence for a goal-based cognitive rehabilitation intervention for this population and support its use in routine care.

## Notes

Supplement 1.
Trial Protocol and Statistical Analysis Plan

Supplement 2. **eTable 1.** Patient and public involvement and engagement (PPIE) in the CICERO randomized clinical trial **eTable 2.** Results of regression models run as a sensitivity analysis on a complete case dataset **eTable 3.** Levels of missing data per measure **eTable 4.** Results from regression analysis including readiness, difficulty, and importance **eTable 5.** Cognitive rehabilitation strategies used in the CICERO randomized clinical trial **eAppendix.** Adapted version of the Bangor Goal-Setting Interview (BGSI)

Supplement 3.
Data Sharing Statement
