# Supplementary material for: Cognitive Rehabilitation and Functional Outcomes in Long COVID–Related Cognitive Impairment: A Randomized Clinical Trial
Source: JAMA Netw Open. 2026 Jul 1;9(7):e2620687. doi: 10.1001/jamanetworkopen.2026.20687 (PMC13324862; doi:10.1001/jamanetworkopen.2026.20687)
Supplement: Supplement 1. — Trial Protocol and Statistical Analysis Plan [file jamanetwopen-e2620687-s001.pdf]

Formatted: Numbering: Continuous

Cognitive Impairment in Long Covid: PhEnotyping and RehabilitatiOn (CICERO)

Chief Investigator:
Dr Dennis Chan, Institute of Cognitive Neuroscience, UCL

Co-Investigators:

Dr Aida Suarez Gonzalez, Dementia Research Centre, UCL

Dr Zoe Hoare, University of Bangor

Dr Nathan Bray, University of Bangor

Dr Jason Lim (public-patient initiative lead)

Professor Chris Kipps, University Hospital Southampton NHS Trust

Dr Ross Dunne, Greater Manchester Mental Health NHS Trust

Sponsored by:
University College London (UCL)

Protocol version 1.13
20/12/2024

R&D / Sponsor Reference Number(s): EDGE 143067
IRAS Project ID: 302920

Study Registration Number:
UCL Data Protection Number – Z6364106 2021 09 43

PROTOCOL VERSIONS

Version Stage	Versions No	Version Date	Protocol updated & finalised by;	Appendix No detail the reason(s) for the protocol update
Current	1.13	20/12/2024	Dr Dennis Chan	Study extension
Previous	1.12	10/10/2024	Dr Dennis Chan	Study extension.
Previous	1.11	01/05/2024	Dr Dennis Chan	Clarifications of study details and recruitment for sub-studies.
Previous	1.10	08/03/2024	Dr Dennis Chan	Study extension, recruitment extension, withdrawal of research site, updates to sub-studies.
Previous	1.9	09/05/2023	Dr Dennis Chan	Updated study dates (extension)
Previous	1.8	23/04/2023	Dr Dennis Chan	
Previous	1.7	13/03/2023	Dr Dennis Chan	
Previous	1.6	03 Jan 2023	Dr Dennis Chan	
Previous	1.5	16 Oct 2022	Dr Dennis Chan<br>UCL JRO	
Previous	1.4	26 Aug 2022	Dr Dennis Chan<br>UCL JRO	
Previous	1.3	17 May 2022	Dr Dennis Chan<br>UCL JRO	
Previous	1.2	27 April 2022	Dr Dennis Chan<br>UCL JRO	
Previous	1.0	17 Feb 2022	Dr Dennis Chan<br>UCL JRO	

DECLARATIONS

The undersigned confirm that the following protocol has been agreed and accepted and that the investigator agrees to conduct the study in compliance with the approved protocol and

will adhere to the Research Governance Framework 2005 (as amended thereafter), the Trust Data & Information policy, Sponsor and other relevant SOPs and applicable Trust policies and legal frameworks.

I (investigator) agree to ensure that the confidential information contained in this document will not be used for any other purposes other than the evaluation or conduct of the clinical investigation without the prior written consent of the Sponsor.

I (investigator) also confirm that an honest accurate and transparent account of the study will be given; and that any deviations from the study as planned in this protocol will be explained and reported accordingly.

Chief Investigator:

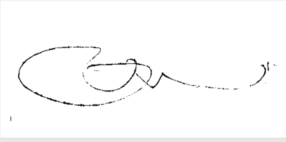

Signature:.. Date....19../....08../....2022...

Print Name(in full):.....Dr Dennis Chan.........................

Position:..Principal Research Fellow, UCL Institute of Cognitive Neuroscience...................

On behalf of the Study Sponsor:

Signature: Date 17/2/2022

Print Name(in full):......Dr Maurice Griffin

Position: Sponsorship Officer, Joint Research Office, University College London

STUDY SUMMARY

**Identifiers**	
IRAS Number	302920
REC Reference No	22/EE/0074
Sponsor Reference No	143067
Other research reference number(s) (if applicable)	UCL Data Protection Number – **Z6364106 2021 09 43**
Full (Scientific) title	**Cognitive Impairment in Long Covid: PhEnotyping and RehabilitatiOn (CICERO)**
Health condition(s) or problem(s) studied	Long Covid
Study Type i.e. Cohort etc	Intervention
Target sample size	120-200
**STUDY TIMELINES**	
Study Duration/length	2 years
Expected Start Date	January 2023
End of Study definition and anticipated date	end of Februart 2025
Key Study milestones	first patient recruitment – workstream 1<br>last patient recruitment - workstream 1<br>first patient recruitment – workstream 2<br>last patient recruitment – workstream 2<br>last patient follow up
**FUNDING & Other**	
Funding	NIHR
Other support	Recruitment, testing, and rehab will be at the three clinical sites (Princess Royal Hospital Haywards Heath, Southampton Hospital, Greater Manchester Hospitals).<br>Statistical analyses will be done at the North Wales Clinical Trials Unit, Bangor. Health economic analyses will be done at the Centre for Health Economics and Medicines Evaluation, Bangor University.<br>Trial coordination is at the main clinical site under Dr Chan (Princess Royal Hospital Haywards Heath).<br>MRI scanning will be done at the Clinical Imaging Sciences Centre, University of Sussex.
**STORAGE of SAMPLES (if applicable)**	N/A
Human tissue samples	N/A
Data collected / Storage	Results of cognitive tests and MRI data will be collected and stored pseudonymously on UCL servers.
**KEY STUDY CONTACTS**	Full contact details including phone, email and fax numbers
Chief Investigator	Dr Dennis Chan<br>Dennis.chan@ucl.ac.uk<br>Principal Research Fellow

	MB BChir PhD MD FRCP<br>UCL<br>Institute of Cognitive Neuroscience<br>7 Alexandra House<br>Queen Square, London<br>WC1N 3AR
Other study personnel	Dr Aida Suarez Gonzalez<br>Dementia Research Centre, UCL<br>aida.gonzalez@ucl.ac.uk<br><br>Dr Zoe Hoare<br>North Wales Organisation for Randomised Trials in Health<br>University of Bangor<br>z.hoare@bangor.ac.uk<br><br>Dr Nathan Bray<br>North Wales Organisation for Randomised Trials in Health<br>University of Bangor<br>n.bray@bangor.ac.uk<br><br>Professor Chris Kipps<br>Department of Neurology<br>University Hospitals Southampton NHS Trust<br>Christopher.kipps@soton.ac.uk<br><br>Dr Ross Dunne<br>Department of Psychiatry<br>Greater Manchester Mental Health NHS Trust<br>Ross.Dunne@gmmh.nhs.uk<br><br>Dr Jason Lim (PPI lead)<br>jasonkylim@msn.com<br><br>Trial Statistician:<br><br>Rachel Evans<br>NWORTH Clinical Trials Unit<br>Bangor University<br>r.evans@bangor.ac.uk

KEY ROLES AND RESPONSIBILITIES

SPONSOR: The sponsor is responsible for ensuring before a study begins that arrangements are in place for the research team to access resources and support to deliver the research as proposed and allocate responsibilities for the management, monitoring and reporting of the research. The Sponsor also has to be satisfied there is agreement on appropriate arrangements to record, report and review significant developments as the research proceeds, and approve any modifications to the design.

FUNDER: The funder is the entity that will provide the funds (financial support) for the conduction of the study. Funders are expected to provide assistance to any enquiry, audit or investigation related to the funded work.

CHIEF INVESTIGATOR (CI): The person who takes overall responsibility for the design, conduct and reporting of a study. If the study involves researchers at more than once site, the CI takes on the primary responsibility whether or not he/she is an investigator at any particular site.

The CI role is to complete and to ensure that all relevant regulatory approvals are in place before the study begins. Ensure arrangements are in place for good study conduct, robust monitoring and reporting, including prompt reporting of incidents, this includes putting in place adequate training for study staff to conduct the study as per the protocol and relevant standards.

The Chief Investigator is responsible for submission of annual reports as required. The Chief Investigator will notify the RE of the end of the study, including the reasons for the premature termination. Within one year after the end of study, the Chief Investigator will submit a final report with the results, including any publications/abstracts to the REC.

PRINCIPAL INVESTIGATOR (PI): Individually or as leader of the researchers at a site; ensuring that the study is conducted as per the approved study protocol, and report/notify the relevant parties – this includes the CI of any breaches or incidents related to the study.

KEY WORDS

Long Covid
Cognitive impairment
Cognitive rehabilitation

LIST OF ABBREVIATIONS

CV19	Covid-19
MRI	Magnetic resonance imaging

TABLE OF CONTENTS

1 Lay summary
2 Scientific summary
Background and Aim
Methods
3 Research questions and objectives
4 Background
5 Experimental design and methods
5.1 Plan of investigation
6 Statistical analysis
7 STUDY SCHEDULE
8 CONSENT
9 ELIGIBILITY CRITERIA
9.1 Inclusion Criteria
9.2 Exclusion Criteria
10 RECRUITMENT
11 PATIENT AND PUBLIC INVOLVEMENT (PPI)
12 FUNDING AND SUPPLY OF EQUIPMENT
13 DATA HANDLING AND MANAGEMENT
14 PEER AND REGULATORY REVIEW
15 ASSESMENT AND MANAGEMENT OF RISK
16 RECORDING AND REPORTING OF EVENTS AND INCIDENTS
16.1 Definitions of Adverse Events
16.2 Assessments of Adverse Events
16.3 Recording adverse events
16.4 Procedures for recording and reporting Serious Adverse Events
16.5 Serious Adverse Events that do not require reporting
16.6 Reporting Urgent Safety Measures
16.7 Protocol deviations and notification of protocol violations
16.9 Trust incidents and near misses
17 MONITORING AND AUDITING
18 TRAINING
19 INTELLECTUAL PROPERTY
20 INDEMNITY ARRANGEMENTS
21 ARCHIVING
22 PUBLICATION AND DISSEMINATION POLICY

23. Covid-19 safety precautions
24. References
25. APPENDICES

1 Lay summary

Cognitive impairment is increasingly recognised as a major component of long Covid, and is estimated to be present in 25-75% of affected individuals. This impairment impacts quality of life and the loss of functional ability has major consequences for affected people, their families and the wider economy given people's difficulty in returning to work.

We propose a two-stage study for investigation and treatment of "cognitive Covid".

Stage 1 will determine those aspects of cognitive function that are particularly affected in cognitive Covid and the severity of the impairment. We will also explore the relationship between cognitive impairment and other aspects of long Covid, namely fatigue, anxiety, depression and sleep disturbance. MRI scanning will be used to measure brain structure and connectivity, to identify the brain networks affected in cognitive Covid that may underpin the cognitive dysfunction.

Stage 2 will focus on helping people recover from cognitive Covid. This will involve use of rehabilitation strategies aimed at improving function in those cognitive functions identified in Stage 1 as being most affected, and assessing the benefit of rehabilitation on quality of life and people's ability to return to everyday function. These strategies will be co-produced in collaboration with a group of people living with cognitive Covid. At the end of Stage 2 we will produce a freely available "Covid-19 Cognitive Recovery Guide" for affected people, their close contacts and clinicians.

In conclusion, cognitive impairment is frequently observed in long Covid but at present little is understood about its nature, or how it can be treated. The sheer scale of the CV19 pandemic makes this a top priority unmet need for healthcare worldwide. The aim of this study is to meet this need and to deliver a treatment plan for affected people which will help them return to normal life and working ability.

2 Scientific summary

Background and Aim

Cognitive impairment is recognised as a major component of long Covid, present in 25-75% of affected individuals, but little is known about the nature of this impairment, or how it can be treated. The associated loss of functional ability has major consequences for affected people, their families and the wider economy given the problems caused in terms of return to work. The aim of this study is to determine the nature of the cognitive impairment and deliver cognitive rehabilitation to help a return to normal life.

Methods

In Workstream 1 (n=80) we will establish the phenotype of cognitive Covid. First, it will identify aspects of cognitive function that are most affected. The test battery will include traditional cognitive tests, and encompassing all principal domains, including memory, attention and executive function, all reported to be affected after CV19 infection. Given emerging evidence that the medial temporal lobe (MTL) is vulnerable to direct infiltration by the neurotropic SARS-CoV-2 virus via neural pathways from the olfactory bulb, we will additionally use the Neotiv suite of app-based cognitive tests https://www.neotiv.com/en to probe different memory processes (pattern completion, face-name association, scene recognition and mnemonic discrimination) subserved by the MTL and not adequately covered with traditional tests. These tests are used for research purposes only and are not used for clinical diagnostic purposes. We will also include an online battery of cognitive tests developed on Gorilla, https://gorilla.sc/. These tests probe several cognitive domains, with a focus specifically on executive function and attention. The addition of assessment 3 allows for a more comprehensive assessment of cognitive functioning. The Gorilla tests are used for research purposes only, not as clinical tools.

Second, we will determine how the cognitive impairment in long Covid is modulated by other aspects of long Covid, namely fatigue, anxiety/depression and sleep disturbance. Third, we will identify the imaging correlates of cognitive Covid, using multimodal MRI. MRI markers of brain health such as brain volume, microstructural integrity, cerebral blood flow and structural and functional connectivity will provide crucial information on the potential pathological underpinnings of cognitive Covid.

Workstream 2 (n = 120) will use cognitive rehabilitation to help recovery from cognitive Covid, building on previous work showing that cognitive rehabilitation improves cognitive and functional outcomes in patients with acquired cognitive impairment. The rehabilitation programme will focus on those cognitive domains found in Workstream 1 to be most affected, and will include remediation, compensatory and adaptive strategies. The programme will be co-produced in collaboration with a group of people living with cognitive Covid, led by a PPI co-investigator. At the end of Workstream 2 we will produce a freely available “Covid-19 Cognitive Recovery Guide” for affected people, their close contacts and clinicians.

A randomised controlled trial will compare the intervention (cognitive rehabilitation) against standard of care (management of fatigue, anxiety and sleep), with the primary outcome being participant-set goals (measured using the Bangor Goal Setting Interview) at three months. Secondary outcome measures will be measures of cognition and quality of life at six months. With a very conservative effect size of 0.7 and estimated attrition rate of 25%, a sample size of 118 (50:50 randomisation) is required.

The health economic benefits of treatment will be assessed in terms of health resource utilisation, primary (cost per QALY) and secondary cost-utility analyses and generation of cost-effectiveness acceptability curves.

3 Research questions and objectives

This proposal comprises two workstreams. Workstream 1 aims to phenotype cognitive Covid-19, while Workstream 2 will develop and test the effectiveness of a neuropsychological rehabilitation intervention to support recovery from cognitive Covid

3.1 Research questions Workstream 1

- What is the phenotype of 'cognitive' Covid?
  - What aspects of cognition are primarily affected in cognitive Covid and what is the range of severity?
- What is the inter-relationship between cognitive impairment and other core features of long Covid, ie fatigue, sleep disturbance, anxiety and depression?
- What are the MRI correlates of cognitive Covid, measured in terms of brain structure and connectivity?
- What is the effect of the CV19 vaccine on cognitive symptomatology?
- Does cognitive impairment in long Covid correlate with molecular markers of inflammation or neurodegeneration?

3.2 Research questions Workstream 2

- Can neuropsychological rehabilitation improve the cognitive and functional outcomes of people with cognitive Covid?
- What is the incremental cost-effectiveness of CIVIC-Rehab compared to standard clinical care for cognitive Covid?
- What is the cost per quality-adjusted life year (QALY) of CIVIC-Rehab compared to standard clinical care for cognitive Covid, and does this fall below the NICE threshold of £20,000 to £30,000 per QALY?

3.3 Objectives

Workstream 1

- To establish the phenotype of cognitive Covid in terms of cognitive domains affected and the association with other symptoms of long Covid
- To identify neuroimaging correlates of cognitive Covid
- To identify molecular correlates of cognitive Covid

Workstream 2

- To develop and test the effectiveness of a neuropsychological rehabilitation intervention programme for cognitive Covid (CIVIC-Rehab) to deliver improvements in clinically relevant outcomes including quality of life and functional ability
- To investigate the incremental cost-effectiveness of the CIVIC-Rehab intervention compared to standard clinical care, using multiple measures of effect.

4 Background

4.1 What is the problem being addressed?

This study addresses the problem of “cognitive Covid”, namely the persisting cognitive impairment that is increasingly recognised as a major component of long Covid[1] and separate to the long term cognitive sequelae of acute CV19 neurological disorders such as stroke and encephalitis. Estimates of the prevalence of cognitive impairment in long Covid vary from 20-75%[2,3]. However, while initial studies indicate that episodic memory, attention and executive function are among the most affected cognitive domains, very little is currently known about the phenotypic range of cognitive Covid, its biological correlates or the degree to which the cognitive impairment is modulated by other aspects of long Covid such as fatigue, sleep disturbance and anxiety.

It is increasingly accepted that cognitive impairment in long Covid reduces quality of life and compromises the ability to undertake everyday activities including work. Given the enormous number of people infected with CV19 worldwide, this represents a new health problem of the highest priority for affected individuals as well as national health services and economies.

We will address this problem by delivering both a detailed phenotypic understanding of cognitive Covid and a neuropsychological rehabilitation programme aimed at mitigating its effects on everyday function.

4.2. Why is this research important in terms of improving the health and/or wellbeing of the public and/or to patients and health care services?

Detailed profiling of cognitive Covid is a necessary first step before development and implementation of rehabilitation programmes targeting those cognitive domains found to be preferentially affected. Given the likely heterogeneity within the cognitive phenotype, to deliver maximum benefit to individuals the rehabilitation will be structured in modular fashion. The core programme will focus on those cognitive domains found to be most commonly and severely affected, around which optional add-on programmes will address other cognitive impairments found in this study to contribute to the functional impairments associated with cognitive Covid.

This work will lead to several important improvements in care. First of all, profiling of cognitive Covid will assist future diagnosis of this condition, particularly in terms of differentiating it from other cognitive disorders including those caused by neurodegenerative disease. Second, determination of the interaction between cognitive impairment and potentially modifiable aspects of long Covid, such as sleep disruption and anxiety, will identify those aspects of cognitive function which can be improved with therapeutic interventions targeting these modifying factors. Third, the provision of neuropsychological rehabilitation strategies will deliver better clinical outcomes for patients while health services

will additionally gain from provision of treatment plans suitable for widespread implementation.

4.3 Review of existing evidence – How does the existing literature support this proposal?

Long Covid is a new condition and as such there is no prior information concerning the nature of cognitive impairment within the disorder, its underlying biological basis or potential treatability. However, there is considerable evidence to support the hypothesis that cognitive dysfunction is a major component of long Covid and that this may respond to neuropsychological rehabilitation, resulting in improved functional outcomes.

4.3.1 Cognitive impairment after acute CV19 infection

Given the relative recency of the CV19 pandemic, the majority of available data on cognitive impairment post-infection relates to cases observed during and shortly after acute infection. A meta-analysis of over 3,500 cases of coronavirus infections (SARS, MERS and CV19) showed that cognitive impairment was present in a third of cases during admission[4], with 33% of CV19 cases having executive dysfunction at discharge[5]. Ongoing cognitive impairment beyond the acute stage is underlined by another study showing persisting cognitive dysfunction in over 75% of cases with mild-moderate acute disease. Work to date indicates that the cognitive domains primarily affected are episodic memory, attention and executive function[2,5-7] with the cognitive impairment being independent of age, gender and prior medical conditions[8].

The notion that cognitive Covid is a major and distinct aspect of long Covid is reinforced by observations that the occurrence of cognitive impairment is unrelated to prior ITU admission and is not associated with fatigue, depression or severity of acute inflammation[9]. Such data indicate that cognitive impairment occurs even after milder infections, independent of comorbidities or stress reactions. The potential negative impact on working age individuals, and in turn personal and national economies, is underscored by a UK study showing that dementia-like cognitive impairment was found in 26% of affected individuals, half of whom were working age[10].

4.3.2 Vulnerability of the central nervous system (CNS) to CV19

The prevailing hypothesis is that the vulnerability of certain physiological systems to SARS-CoV-2 relates to expression of the ACE2 receptor on cell surfaces, onto which viral proteins bind and obtain cellular entry. In the CNS the ACE2 receptor is highly expressed in neurons, astrocytes, oligodendrocytes and endothelial cells. Regionally, high ACE2 receptor concentrations are found in the olfactory bulb, substantia nigra, middle temporal gyrus, and

posterior cingulate gyrus[11]. Recent work has confirmed the ability of SARS-CoV-2 to invade the brain from the periphery, with entry via the olfactory neural-mucosa interface[12]. This provides the means by which SARS-CoV-2 can infiltrate brain regions receiving direct projects from the olfactory bulb, including the hippocampus and other medial temporal lobe regions. Given the role of these regions in memory, the widely reported memory impairment in cognitive Covid may relate in part to direct viral infection of these regions.

4.3.3. Evidence of efficacy of cognitive rehab in other disorders

While there is a lack of any evidence base on the use of neuropsychological rehabilitation in individuals affected by the previous coronaviruses such as SARS, there is extensive experience in managing the cognitive sequelae of other brain infection disorders such as HIV[13] and infectious encephalitis[14]. This in turn builds on more than 40 years of research and clinical practice on cognitive rehabilitation in people affected by neurological conditions[16-19].

Restorative (e.g., retraining) or compensatory (e.g., strategies to adapt to memory impairment) are helpful in managing deficits in memory, attention and executive function arising from a wide range of neurological aetiologies[20-23]. Currently, rehabilitation therapists can resort to a wide range of tools, strategies and aids to treat cognitive impairment. In a systematic review of cognitive rehabilitation for infectious encephalitis, 5/9 studies showed positive changes in memory[14]. Cognitive training and rehabilitation can also lead to gains in executive function and memory in people with cognitive deficits secondary to HIV, and these gains are maintained over time[13]. Studies of other neurological disorders also show benefits of training, with improved executive function in patients with multiple sclerosis who undertake therapeutic training[24].

In summary, work undertaken in patients with aetiologies and cognitive deficits similar to those described in cognitive Covid shows benefit from cognitive rehabilitation therapies. This informs the rationale for the current study.

5 Experimental design and methods

5.1 Plan of investigation

5.1.1 Participant recruitment

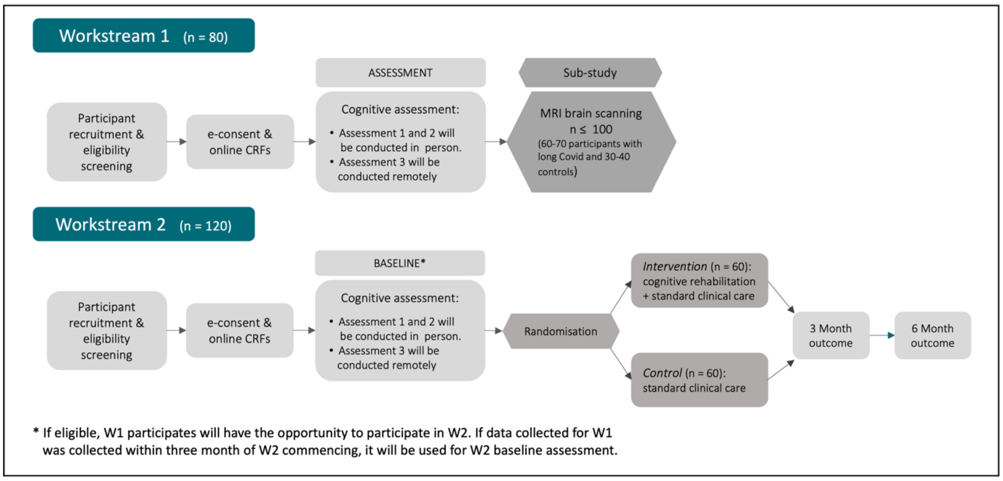

Figure 1. Trial flowchart including sample size

Workstream 1

80 participants will be recruited from three clinical sites, namely the Cognitive Disorders Clinic, University Hospitals Sussex NHS Trust (PI *Chan),* representing one of the first clinics nationwide to offer a dedicated diagnostic and therapeutic service for cognitive Covid, Southampton General Hospital (PI *Kipps*), and Greater Manchester Mental Health NHS Foundation Trust (*PI Dunne*). As part of a substudy, 100 participants (60-70 with long Covid and 30-40 controls) will undergo MRI scanning at the University of Sussex.

Participants will be offered reimbursement of up to £20 for each study visit.

Workstream 2

For the RCT 120 participants (for justification of this sample size, see power calculation below) will be recruited from the same clinics. If eligible participants in WS1 can take part in WS2.

5.2.1 Work Stream 1: Phenotyping Cognitive Covid

Baseline demographic information will include: age, gender identity, sexual orientation, registered sex at birth, medical and drug history, ethnicity, years of formal education, socio-economic status, disability, and language. We will also acquire information on i) temporal relationship between acute CV19 symptoms and onset of cognitive problems, ii) temporal relationship between onset of other long Covid symptoms and onset of cognitive problems, iii) CV19 vaccination history and reported effect of vaccine (if any) on cognition.

Participants will complete several questionnaires including:

- Quality of life (EQ-5D-5L)

- Life Space Assessment
- Social Functioning (SF-DEM)
- Instrumental Activities of Daily Living (IADL) Scale
- Generalised Anxiety Disorder Assessment (GAD-7)
- Patient Health Questionnaire (PHQ-8)
- Chalder Fatigue Scale
- Pittsburgh Sleep Quality (PSQI)
- DePaul Symptom Questionnaire – Post-Exertional Malaise (DSQ-PEM).

Cognitive function will be assessed using three complementary assessments (expanded below), to maximise understanding of cognitive phenotype.

Assessment 1 will be a battery of cognitive tests currently used in clinic for the diagnosis of cognitive disorders probing working memory, attention, cognitive speed and executive function, representing domains primarily affected after CV19 infection.
Specifically, assessment 1 includes:

- The Repeatable Battery for the Assessment of Neuropsychological Tests (RBANS)
- The Delis-Kaplan Executive Function System: The Trail Making Test (Condition 2: Number Sequencing & Condition 4: Number-Letter Switching)
- The Delis-Kaplan Executive Function System: Colour-Word Interference
- The Delis-Kaplan Executive Function System: Phonemic Fluency
- Wechsler Adult Intelligence Scale III: Digit Span
- Test of Premorbid Functioning (TOPF)

Assessment 2 will involve the use of an online battery of cognitive tests. The first set of tests were designed in-house by Professor Paul Burgess of the UCL Institute of Cognitive Neuroscience, and developed on Gorilla, https://gorilla.sc/. These tests probe speed of information processing, executive function and attention and are used in CICERO given these are the cognitive domains most commonly reported by patients as being affected in long Covid. The second online cognitive test is the 4 Mountains Test (4MT). It is a brief assessment of allocentric spatial memory, representing a key function of the hippocampus. These tests are used for research purposes only, not as clinical tools.

Assessment 3 will involve use of the Neotiv suite of app-based cognitive tests https://www.neotiv.com/en . These tests probe several aspects of episodic memory, representing different processes and functions of the hippocampus and entorhinal cortex (pattern completion, scene recognition and mnemonic discrimination) that are not adequately covered with traditional cognitive tests. These additional tests are applied to reflect i) emerging evidence that the entorhinal cortex and hippocampus are particularly vulnerable to direct viral infiltration, ii) recent work showing entorhinal-hippocampal dysfunction in people with long Covid in the absence of any subjective reporting of impairment of episodic memory.

Sub-study - UK normative data for Neotiv

There will be a substudy which will recruit 20 female, 20 male healthy participants (N=40) matching the demographic characteristics of the CICERO study. At present, the data for Neotiv has been normed in a German population. This substudy will therefore allow for the collection of UK specific normative data relevant to the CICERO sample. Healthy age-matched controls for the neotiv sub-study will be recruited via the UCL participant databases. We also intend to recruit family members of participants involved in Stage 1 and Stage 2 where eligible, as well as participants from databases associated with our collaborating research sites. Recruiting from a variety of sources will ensure access to the relevant age group and an ethnically diverse population. Testing will take place at University Hospitals Sussex NHS Trust and UCL.

Sub-study - Normative data for 4 Mountains Test

The 4 Mountains Test (4MT) is a short computerised test of spatial memory. It is included as part of the cognitive assessment detailed above. At present, there is no normative data for the 4MT. This substudy will therefore allow for the collection of UK specific normative data relevant to the CICERO sample. This substudy intends to recruit 25 female, 25 male healthy participants (N=50) matching the demographic characteristics of the CICERO study. Healthy age-matched controls for the 4MT sub-study will be recruited via the UCL participant databases. We also intend to recruit family members of participants involved in Stage 1 and Stage 2 where eligible, as well as participants from databases associated with our collaborating research sites. Recruiting from a variety of sources will ensure access to the relevant age group and an ethnically diverse population. Testing will take place at University Hospitals Sussex NHS Trust and UCL.

Sub-study – Healthy Controls

This sub-study encompasses the assessments from both the Neotiv and 4 Mountains Test sub-studies, while incorporating additional cognitive tests. It aims to gather UK-specific normative data pertinent to the CICERO sample by integrating the assessments conducted in the preceding sub-studies. This substudy intends to recruit 25 female, 25 male healthy participants (N=50) matching the demographic characteristics of the CICERO study.

Participants will complete the 4 Mountains Test (4MT), which is a short computerised test of spatial memory. They will also complete a selection of cognitive tests designed in-house by Professor Paul Burgess of the UCL Institute of Cognitive Neuroscience, and developed on Gorilla, https://gorilla.sc/. Some participants will be invited to complete the cognitive assessment on ‘neotivTrials’ app.

Healthy age-matched controls will be recruited via UCL participant databases. We also intend to recruit family members of participants involved in Stage 1 and Stage 2 where eligible, as well as participants from databases associated with our collaborating research sites. Recruiting from a variety of sources will ensure access to the relevant age group and an ethnically diverse population. Testing will take place at University Hospitals Sussex NHS Trust and UCL.

Sub-study – Cognitive rehabilitation workshop for control group participants

Control group participants will be invited to participate in an online workshop with a focus group outlining the strategies used in neuropsychological rehabilitation of cognitive impairment in long Covid (“cognitive Covid”). The workshop has dual objectives: firstly is to explore the experiences of individuals living with cognitive Covid and their coping mechanisms. Secondly, to provide an educational workshop on the neuropsychological strategies used in cognitive rehabilitation that may be used to cope with cognitive difficulties associated with long Covid syndrome. The session will be recorded for analysis purposes and direct quotations from participants (de-identified) may be used in publications. The expected duration of the workshop is 1.5 hours in a single session Before the start of the workshop, we will give participants the option to keep their cameras off for the duration of the workshop, if they do not wish to be video recorded. We will video-record the workshop with an automatic transcript on MS Teams. Within 72 hours after the workshop, two research assistants will compare the automatic transcript against the video recording. Afterwards, they will upload the transcript to Data Safe Haven (DSH), and the video recording will be deleted.

Data collection

Participants will give consent and complete case report forms (CRFs), including demographic information and questionairres, via a secure REDcap weblink which will save data directly to UCL’s data safe haven . In the event that participants are not able to complete the electronic consent and CRF, they will be able to attend clinic to complete a paper-based consent form and CRF. Cognitive assessment 1 and 2 will be completed in clinic, and cognitive assessment 3 will be completed remotely.

Data analysis

Within the cross-sectional dataset investigation of the relationships between cognitive function, fatigue, sleep disturbance, anxiety/depression and social isolation will be undertaken using linear mixed models accounting for demographic factors.

5.2.1.1 MRI substudy

We will scan the brains of 100 participants on a Siemens 3T Prisma scanner at the Clinical Imaging Sciences Centre, University of Sussex. Of the 100 participants scanned, 60-70 will be long Covid patients recruited primarily from the Sussex memory clinic as part of Workstream 1. The remaining participants will be age and sex matched healthy controls (n=30-40). The scan protocol will include the following sequences:

- T1-weighted (whole brain and regional volumes)
- FLAIR (white matter hyperintensity volume, a measure of vascular damage)
- Diffusion tensor imaging (structural connectivity)
- NODDI (microstructural integrity)
- Resting state fMRI (functional connectivity)
- ASL (cerebral blood flow and blood brain barrier permeability)

- Quantitive Susceptibility Mapping (molecular composition and cellular architecture of the tissue)

The aim of this MRI substudy is to determine the neuroimaging correlates of cognitive impairment in long Covid.

MRI data collection

Cognitive testing and MRI scanning will take place once, at study entry, noting also the interval between the date of acute infection and test date.

MRI data analysis

MRI analyses will be undertaken using the freely available, open-source tools. These tools include NiftySeg (https://github.com/KCL-BMEIS/NiftySeg), NiftyFit (https://discovery.ucl.ac.uk/id/eprint/1489639/), Matlab's NODDI toolbox (http://mig.cs.ucl.ac.uk/index.php?n=Tutorial.NODDImatlab), ExploreASL (https://sites.google.com/view/exploreasl) and Freesurfer (https://surfer.nmr.mgh.harvard.edu). Using these software packages, we will be able to assess the impact of cognitive Covid on various measures of brain health, such as whole brain and regional volumes, microstructural integrity, structural and functional connectivity and cerebral blood flow.

5.2.2 Work Stream 2: Use of neuropsychological rehabilitation to aid recovery from cognitive Covid

The second workstream will develop a suite of phenotype-specific and evidence-based neuropsychological rehabilitation packages targeted at the cognitive domains primarily affected in cognitive Covid, using functional and quality of life outcomes to measure treatment efficacy. This collection of strategies will be informed by outcomes from workstream 1 and co-produced in collaboration with a group of people living with cognitive Covid-19 (we will refer to this group as Experts by Experience).

Intervention Development.

We will develop phenotype-specific neuropsychological rehabilitation plans targeting the predominantly affected cognitive domains as uncovered in Workstream 1, with methods informed by past work on use of neuropsychological rehabilitation to alleviate cognitive deficits arising from CNS infection. To allow for anticipated heterogeneity in the severity and range of cognitive impairment in people with long Covid, different types of cognitive intervention will be employed, including remediation techniques (e.g. improving attention by lengthening the time to complete simple tasks), compensatory and adaptive strategies (e.g. use of memory notebooks and mnemonics). Our neuropsychological rehabilitation plans for Covid-19 will follow the 2013 NICE guidelines on Stroke rehabilitation (section 1.4. Cognitive functioning), 2013 SIGN guidelines on Brain Injury Rehabilitation and evidence

provided by recent systematic reviews[25,26] SIGN advises on the approach to address the rehabilitation of memory, attention and executive function and recommends the use of therapies such as meta-cognitive strategies (e.g. problem-solving, goal-management and strategic reasoning to treat executive dysfunction, task-specific training for attentional deficits and the use of external aids (e.g. digital diaries) and internal strategies (e.g. use of visual imagery) for mild-moderate memory problems. Likewise, the NICE guidelines recommend that attention training is focused on relevant functional tasks and training on the use of internal strategies to encode information to treat memory problems, as well as environmental strategies (e.g. environmental prompts) and use of external aids. The goal of the intervention trialled in WS2 is to reduce disability due to 'cognitive Covid' and restore a level of function as similar as possible to level pre-infection.

The interventions will be delivered in 10 individual sessions of 1 hour duration over a period of 3 months (1 session per week approximately). In the first session relevant goals will be identified, alongside with a suite of strategies adjusted to the individual needs based on: 1) existing strategies already tried by the individual and found successful, 2) information from WS1 about the individual's profile of cognitive impairment (e.g. dominant dysexecutive function vs dominant amnestic impairment) and 3) personal preferences.

Each therapy session will include, among others: training on restorative strategies to support the learning of new information., modelling of specific strategies and skills, improving the efficiency of existing strategies and improvement or compensation of attentional and concentration disorders. Some of the strategies that will be implemented: goal management training, chunking, pacing, environmental modifications, dual-task training and multimodal encoding. Participants will receive tasks to complete in between sessions and apply in everyday situations. Strategies will be refined and adjusted over sessions as needed.

At the end of the data collection, participants in the control group will be invited to an online workshop where they will receive information about the study findings and about how to use for their benefit the rehabilitation strategies and techniques found successful in the intervention group. Anonymised feedback collected from their participation in these meetings will inform our creation of educational material and tutorials that will be eventually made freely available on NHS websites.

Following work completion, we will produce a Covid-19 Cognitive Recovery Guide (CICERO-Rehab). This is a manual for future clinical usage, describing a step-by-step approach to the rehabilitation to this patient group as developed in the study. The guide will include the approach for adapting the cognitive rehabilitation intervention to different cognitive Covid-19 phenotypes.

5.2.2.1 Trial design

This will be an individually randomised parallel group controlled trial, comparing the study intervention (cognitive rehabilitation) with the clinical standard of care (current clinical management pathways for long Covid, including treatment for fatigue, respiratory disorders, sleep, anxiety and depression).

5.2.2.2 Participant flow

Post-consent participants will undergo baseline assessment before randomisation to receive either neuropsychological rehabilitation or treatment as usual.

5.2.2.3 Outcome measures

The primary outcome will be participant-reported goal attainment at 3 months post-randomisation. This will also be collected at the 6 month post-randomisation follow up. This will be collected using the Bangor Goal Setting Interview (BGSI).

Secondary outcomes will be:

- Cognitive function (tested using the cognitive batteries employed for Workstream 1)
- Quality of life (EQ-5D-5L)
- Life Space Assessment
- Social Functioning (SF-DEM)
- Instrumental Activities of Daily Living (IADL) Scale
- Generalised Anxiety Disorder Assessment (GAD-7)
- Patient Health Questionnaire (PHQ-8)
- Chalder Fatigue Scale
- Pittsburgh Sleep Quality (PSQI)
- DePaul Symptom Questionnaire – Post-Exertional Malaise (DSQ-PEM).
- Client Service Receipt Inventory (CSRI)

5.2.2.4 Health economic analyses

From a public sector, multi-agency perspective we will conduct an economic evaluation to determine the incremental cost-effectiveness of the intervention in comparison to clinical standard of care. The intervention of interest will be a novel neuropsychological rehabilitation intervention programme for cognitive Covid compared to clinical standard of care (i.e. current clinical management pathways for long Covid, including treatment for fatigue, respiratory disorders, sleep, anxiety and depression). We will examime two research questions during the economic evaluation:

1. What is the incremental cost-effectiveness of CICERO-Rehab compared to standard clinical care for cognitive Covid?
2. What is the cost per QALY of CICERO-Rehab compared to standard clinical care for cognitive Covid, and does this fall below the NICE threshold of £20,000 to £30,000 per QALY?

The clinical trial will provide data pertaining to cost, intervention characteristics, outcomes and other health/economic data which will be used during the health economic evaluation. Analysis with complete and well-structured data is preferable but we are often faced with the possibility of missing data and/or skew data resulting from several issues such as incomplete data and patient withdrawal. To handle these issues an appropriate Statistical Analysis Plan (SAP) such as the Bayesian simulation method, an imputation method or complete case analysis will be conducted. Our choice of method will be determined by the quantity and quality of primary data. All relevant data inputs will undergo full validation and verification by the trial team, and uncertainty will be considered within the sensitivity analysis. Discounting of costs and outcomes will not be relevant as the follow-up period is less than 12 months.

Health economic outcome measures

For the primary cost-effectiveness analysis, the primary outcome will be QALYs, derived from the EQ-5D-5L outcome measure. The EQ-5D-5L is a generic, preference based, health-related quality of life (HRQoL) measure. It consists of two parts, a five item questionnaire and a visual analogue scale (EQ-VAS). The EQ-5D-5L questionnaire is scored between 0 and 1, with 1 meaning full HRQoL and 0 death (some scores may fall below 0). Each set of answers on the EQ-5D-5L is assigned a utility score between 0 and 1 based on general population weighting of health states. This measure can then be used to generate QALY estimates by assigning a quality of life weight to the length of time a person is in a given health state.

Secondary cost-effectiveness analyses will be conducted using the Bangor Goal Setting Interview and HADs outcome measures. These will be used to develop incremental cost-effectiveness ratios (ICERs) to express cost-effectiveness in natural units of effect, which will in turn allow comparison with the QALY outcomes. Comparing these outcomes will help to determine relative agreement between the generic QALY outcomes and 23linically relevant outcomes.

Health economic data collection

In order to collect data on the cost of the interventions, health service use and wider costs/benefits to society, we will record study participant primary and secondary care health and social care service use over the follow-up period using a client service receipt inventory (CSRI) to collect service use data from participants, and further triangulated with linked routinely collected data. We will make use of national unit costs and routine hospital data/manufacturers data to cost the intervention and service use. We will also consider the financial implications of presenteeism and absenteeism by calculating the number of participant workdays lost during the follow-up period and triangulating with income data.

Health economic data analysis

We will conduct an economic evaluation at the end of the 3-month follow-up period, based on the following components:

- Conduct a primary cost-utility analysis using the EQ-5D-5L as the measure of utility to generate a cost per QALY estimate.
- Conduct a secondary cost-effectiveness analysis using the relevant trial outcomes (Bangor Goal Setting interview, HADs), and develop incremental cost-effectiveness ratios (ICERs) to express cost-effectiveness.
- For all analyses, we will use bootstrapping to produce cost-effectiveness acceptability curves[30] for comparison with the NICE ceiling of £20,000 to £30,000 per QALY in the UK[31] to communicate to policymakers the probability that the intervention is cost-effective.
- Conduct sensitivity analyses to investigate uncertainty in the data.

The incremental costs and consequences of the treatment arms will be compared and expressed in cost per QALYs where possible. We will use a UK tariff as a source of EQ-5D-5L values for the cost-utility analysis and compare values to UK population norms. In the event that neither intervention is found to have a significant effect on patient outcomes, we will be unable to perform a robust analysis of cost-effectiveness. We will therefore present a cost-consequence analysis, whereby the costs and effect data are presented in a disaggregated manner. Additional cost-effectiveness analyses using the secondary outcomes will be carried out for comparative purposes

We will use both deterministic and probabilistic sensitivity analysis to test uncertainty of findings. Sensitivity analysis is used in economic evaluations to test how sensitive the findings are to basic assumptions used in the economic evaluation model. For instance, the cost of an intervention is to some extent based on assumptions about unit costs and staff time, likewise effects are subject to uncertainty between individuals. By varying these assumptions the stability of findings can be tested, and uncertainty can be accounted for.

Deterministic sensitivity analysis can be either univariate or multivariate, where-by single or multiple parameters may be individually adjusted (within a given range of uncertainty) to test findings of the model. For instance, the cost of the intervention may be incrementally increased or decreased (within given confidence limits) to examine the impact on cost-effectiveness or costs per QALY outcomes. Probabilistic sensitivity analysis assigns a distribution of point estimates to each parameter and randomly selects a single value for each model calculation. By running a number of replications, for instance 5000, an ICER plane can be generated to illustrate the potential variation in cost-effectiveness based on altering basic assumptions about effectiveness and costs.

6 Statistical analysis

Primary analysis of the BGSI will be completed on an ITT basis at the 3 month endpoint using mixed effect models. Stratification variables will be incorporated into the model and other potential factors that could affect the results will be determined a priori and considered for inclusion in the models. All treatment effect estimates will be presented with 95% confidence intervals and 5% level of significance will be used. Analysis of secondary outcomes will follow the same analysis model as the primary analysis where possible. Binary outcomes will be analysed using multi-level logistic regression. Exploratory analysis will be conducted to investigate the effect of phenotypes and comorbidities on the outcome.

Where possible missing data will be minimised however a maximum-likelihood multiple imputation approach will be used with a sensitivity comparison of complete case dataset.The planned analysis will be specified in the statistical analysis plan (SAP), any deviations from the analysis plan will be justified and fully documented in the results report.

7.1 Power calculation

Evidence from the GREAT trial of cognitive rehabilitation in neurodegenerative disease using the same primary outcome Bangor Goal Setting Interview (BGSI) indicated an effect of approximately 0.8 in a population with dementia[18]. This is likely to be an underestimation of the effect in this study as it is likely that the intervention will be more effective in people without neurodegeneration as seen in the population in the GREAT trial.
Assuming a minimally clinically important difference of 2 points (https://www.thecopm.ca/faq/) and a more conservative effect of 0.7 would require SD of the measure to be approximately 2.86. Baseline SD observed in the GREAT trial was 1.74, it is likely that variability in our sample is likely to be larger and therefore we can accommodate an Increase in variability of approximately 65%.
A sample of 88 (44:44) will be required to detect an effect of 0.7 on the BGSI at 3 months with 90% power at a 5% significance level. Incorporating an attrition of 25% will require recruitment and randomisation of 118 participants (59:59).

7 STUDY SCHEDULE

7.1. Enrolment

Participants will be recruited from the cohorts of patients with long Covid and cognitive impairment seen in the Cognitive Disorders Clinics held at University Hospitals Sussex NHS Trust (PI Chan), University Hospitals Southampton NHS Trust (PI Kipps), Greater Manchester Mental Health Trust (PI Dunne). They will initially be approached by the local pIs or associated researchers working at each study site and informed consent for participation will be obtained.

Where necessary, research sites will also recruit from additional sites, including regional Long-Covid services (hosted by Pennine Care NHS Foundation Trust), the “Research For the Future” research database, GP referrals to the research sites and Pennine Care memory clinics, and from tertiary referrals from other COVID clinics (e.g. respiratory clinics). Additionally, University Hospitals Sussex NHS Foundation Trust will recruit from Sussex Community NHS Foundation Trust.This will allow the research sites better access to the target recruitment population, and a more ethnically and socioeconomically diverse and representative population overall. Referrals will be made by staff in the relevant team to the study team at the relevant research site, who will then commence screening and recruitment. With respect to the “Research for the Future” Database, relevant database procedures and policies will be followed. The “Research for the Future” Database will be able to promote CICERO on their website, newsletter, Facebook, or Twitter. The CICERO recruitment poster(s) will also be available for sites to promote the study in clinic. Other research websites, including www.bepartofresearch.nihr.ac.uk and www.clinicaltrials.gov, will be used to promote the study and recruit participants.

These additional recruitment sites, including Pennine Care NHS Foundation Trust and Sussex Community NHS Foundation Trust, will be set up as Participant Identification Centres (PIC), using a model Non-Commercial PIC agreement.

Regarding recruitment to the following sub-studies: Healthy controls sub-study, UK normative data for Neotiv sub-study, and Normative data for 4 Mountains Test sub-study. We will promote the study and recruit from participant databases associated with our collaborating research sites, and UCL participant pools. Poster(s) will be available for sites to promote the sub-studies in clinic, and at UCL. Where necessary, these sub-studies, will also be promoted on the “Be part of research” database.

Regarding recruitment to the Cognitive rehabilitation workshop for control group participants (sub-study). All control group participants from Workstream 2 will be invited to participate in this online workshop.

Participants are free to withdraw from the study at any time.

The end of the study is defined as the last research intervention for the last participant.

7.2 Randomisation Procedure

There will be no randomisation procedure for workstream 1

For workstream 2 randomisation will take place once consent and baseline data collection has been completed. Randomisation will be on a 1:1 allocation (intervention:control) via a secure online system hosted by NWORTH, Bangor University. The system will employ a dynamic adaptive randomisation algorithm. The randomisation will be stratified for centre (London/Sussex/Southampton/Manchester). A member of the research team at UCL will enter the participants details into the web form and perform the randomisation, which will either display directly on screen, if the researcher is unblinded or notification of the randomisation result will be sent to the designated parties to organise ongoing contact.

7.3 Unblinding procedure

There are no blinding requirements in workstream 1.

Due to the nature of the intervention in workstream 2 participants will not be blinded to the allocation they receive. However, every effort will be made to maintain blinding for the researchers collecting data where logistically possible. The Trial Statistician will be blind to allocation until the quantitative analysis, as detailed in the statistical analysis plan (SAP) has been carried out. Once the blinded results have been shared with the study team and the results discussed and interpreted, the unblinded analysis (as detailed in the SAP) such as adherence analysis will be carried out.

7.4 Withdrawal of Subjects

Participants are free to withdraw at any time during the trial without any impact on their future health and care. Participant data collected to the point of withdrawal will be used in the analysis set unless consent for this is specifically withdrawn.

8 CONSENT

If prior to their clinic appointments, patients give consent to be contacted about research studies then the initial approach about this study will be made either by the local PI or a member of his research team.

Informed consent will be documented electronically via a secure REDcap weblink.

9 ELIGIBILITY CRITERIA

These are the same for both Workstreams 1 and 2. However, Workstream 2 has an additional exclusion criteria contingent on findings from baseline cognitive assessment.

9.1 Inclusion Criteria

- Aged between 30 and 60 years
- Evidence of prior CV19 infection
  - *either* positive CV19 PCR
  - *or* positive CV19 antibody test
  - *or* acute symptoms consistent with the recognised core features of acute CV19 infection and post-acute symptoms consistent with the recognised core features of long Covid
- Cognitive impairment persisting more than three months after the acute CV19 infection, defined in terms of subjective reports of cognitive decline post-infection

9.2 Exclusion Criteria

- Cognitive impairment prior to CV19 infection
- Occurrence of acute neurological disorder, such as stroke or encephalitis, that could give rise to cognitive sequelae
- People who are on any medications that are considered by the study investigators to have significant adverse effects on cognition

- A pre-existing major psychiatric or medical disorder that is considered by the study investigators to have potential to affect cognition
- High alcohol intake
- Recreational drug use
- Loss of mental capacity such that the affected individual is unable to give informed consent
- Participants will not be eligible for Workstream 2 if they do not exhibit significant impairment on baseline cognitive assessments, defined as impairment in two or more cognitive domains, as they will not gain from cognitive rehabilitation.
- Participants with pacemakers or other implanted devices, those with metal foreign bodies (e.g. shrapnel from war injuries) and those who have had certain types of surgery will be excluded from the MRI substudy. Although MRI is not known to affect the unborn child, we will also exclude subjects who may be pregnant just to be on the safe side.

10 RECRUITMENT

Patients with cognitive Covid will be recruited prospectively from NHS memory clinics held in Sussex, Southampton and Greater Manchester.
This follows a standard procedure within these clinics. All patients attending clinic are given a form asking about their willingness to be contacted for research. The clinic team keep a record of those that give consent to be contacted.

For those patients who have given consent to be contacted, the research team for this particular study will then provide the study Participant Information Sheet.

Where necessary, research sites will also recruit from additional sites, including regional Long-Covid services (hosted by Pennine Care NHS Foundation Trust), the “Research For the Future” research database, GP referrals to the research sites and Pennine Care memory clinics, and from tertiary referrals from other COVID clinics (e.g. respiratory clinics). Additionally, University Hospitals Sussex NHS Foundation Trust will recruit from Sussex Community NHS Foundation Trust. Overall, this will allow the research sites better access to the target recruitment population, and a more ethnically and socioeconomically diverse and representative population overall. Referrals will be made by staff in the relevant team to the study team at the relevant research site, who will then commence screening and recruitment. With respect to the “Research for the Future” Database, relevant database procedures and policies will be followed. The “Research for the Future” Database will be able to promote CICERO on their website, newsletter, Facebook, or Twitter. The CICERO recruitment poster(s) will also be available for sites to promote the study in clinic. Other research websites, including www.bepartofresearch.nihr.ac.uk and www.clinicaltrials.gov, will be used to promote the study and recruit participants.

These additional recruitment sites, including Pennine Care NHS Foundation Trust and Sussex Community NHS Foundation Trust, will be set up as Participant Identification Centres (PIC), using a model Non-Commercial PIC agreement.

To further ensure the study encompasses people from minority ethnic backgrounds and varying socioeconomic backgrounds we will adjust our recruitment process to both workstreams taking into account age, sex, gender, and ethnicity such that those groups most

likely to be affected by cognitive Covid are well represented. To facilitate this, the following measures will be implemented:

- Preparation of culturally- and linguistically-appropriate recruitment information
- Community outreach
- Cultivating relationships with clinicians caring for diverse groups of patients with long Covid, particularly community CV19 care coordinators

Participants will be included regardless of proficiency in English. For those less proficient then we will seek to find a professional interpreter, and funding for this will be requested of NIHR.

11 PATIENT AND PUBLIC INVOLVEMENT (PPI)

PPI work will be led by Dr Jason Lim, PPI lead, working alongside Dr Suárez-González, who currently leads the PPI element of the Rare Dementia Impact Project (Brotherhood et al., 2019) The Guidance for Reporting Involvement of Patients and the Public (GRIPP) will be use to comply with high quality standards of documentation and reporting of PPI in this study (Staniszewska et al., 2017). The PPI group for this study will consist of a group of 4 people living with ‘cognitive’ Covid-19. They will be identified with the help of PPI co-lead Dr Jason Lim through peer support networks and advocacy groups (https://www.longcovid.org/about). People will be contacted by email or approached face-to-face and provided with written information about the study and their role in it shall they decide to participate. Meetings will take place remotely by videoconference to facilitate logistics and comfort for participants.

Group meetings will take place every three months until month 6 (when the workstream of the intervention starts) and then will become annual (this schedule will be left open to modification to adjust to the needs of the group). We will collect feedback from the PPI groups in the form of written notes and questionnaires and any feedback or participation that takes place by email. The group will be trained at the start of the study on the method of participation (e.g. how to join by video meeting, what the rules of participation will be, how to provide feedback). The PPI team will be particularly heavily involved in workstream 2, taking part in the processes and procedures of this stream (e.g. deciding on the most important elements of the intervention and best way to administer them) and also in the dissemination of outcomes.

12 FUNDING AND SUPPLY OF EQUIPMENT

The study is funded by a competitively awarded research grant to Dr Chan from the National Institute for Health Research, as part of an open call for studies into Long COVID in non-hospitalised individuals. UCL will administer the grant and has a collaboration agreement with the University of Magdeburg, Germany, for usage of their Neotiv app for remote testing of cognitive function. The research grant also funds researchers at the other study sites (University of Bangor, University Hospitals Southampton NHS Trust, Greater Manchester Mental Health NHS Foundation Trust).

13 DATA HANDLING AND MANAGEMENT

The study will include the use of both identifiable data, and pseudonymised data from patients with cognitive Covid. Identifiable information will be stored securely on Data Safe Haven with limited access and will not be used for analysis purposes. Participant identifiable

information will be securely accessed by the clinic and research teams, to contact and schedule appointments with participants. Participant data for analysis will be pseudonymised using a code. The decoding key will be stored on Data Safe Haven. Pseudonymised participant data will be recorded electronically on password protected university laptops and on Data Safe Haven. Data from third parties will be securely transferred using the Data Safe Haven file transfer portal. Pseudonymised MRI data will be transferred from the University of Sussex to UCL servers using UCL XNAT (https://www.ucl.ac.uk/isd/services/research-it/ucl-xnat-service). Data will be maintained on UCL servers to ensure data security.

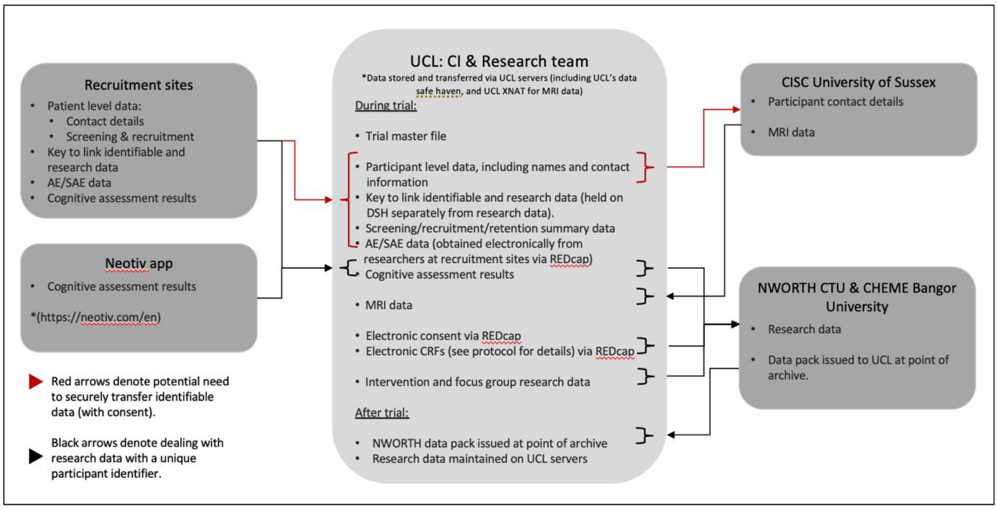

Figure 2. Data flow diagram

14 PEER AND REGULATORY REVIEW

This study has been peer reviewed as part of the NIHR grant application process. The Sponsor has accepted this review as adequate evidence of peer review.

The study was deemed to require regulatory approval from NHS ethics. Each approval will be obtained before the study commences.

15 ASSESMENT AND MANAGEMENT OF RISK

There are no major disadvantages or risks associated with participation.

16 RECORDING AND REPORTING OF EVENTS AND INCIDENTS

16.1 Definitions of Adverse Events

Term	Definition
Adverse Event (AE)	Any untoward medical occurrence in a patient or study participant, which does not necessarily have a causal

	relationship with the procedure involved.
Serious Adverse Event (SAE).	Any adverse event that:<br>• results in death,<br>• is life-threatening*,<br>• requires hospitalisation or prolongation of existing hospitalisation**,<br>• results in persistent or significant disability or incapacity, or<br>• consists of a congenital anomaly or birth defect
*A life- threatening event, this refers to an event in which the participant was at risk of death at the time of the event; it does not refer to an event which hypothetically might have caused death if it were more severe.<br>** Hospitalisation is defined as an in-patient admission, regardless of length of stay. Hospitalisation for pre-existing conditions, including elective procedures do not constitute an SAE.	

16.2 Assessments of Adverse Events

Each adverse event will be assessed for severity, causality, seriousness and expectedness as described below.

16.2.1 Severity

The generic categories below are given for use as a guide.

**Category**	**Definition**
Mild	The adverse event does not interfere with the participant's daily routine, and does not require further procedure; it causes slight discomfort
Moderate	The adverse event interferes with some aspects of the participant's routine, or requires further procedure, but is not damaging to health; it causes moderate discomfort
Severe	The adverse event results in alteration, discomfort or disability which is clearly damaging to health

16.2.2 Causality

The assessment of relationship of adverse events to the procedure is a clinical decision based on all available information at the time of the completion of the case report form.

If a differentiated causality assessment which includes other factors in the study is deemed appropriate, please add/amend the following wording to specify:

It is of particular importance in this study to capture events related to the product application procedure. The assessment of relationship of an adverse event to this/these additional safety issue(s) will also be carried out as part of the study.

The differentiated causality assessments will be captured in the study specific CRF/AE Log and/or SAE form (amend as required).

The following categories will be used to define the causality of the adverse event:

**Category**	**Definition**
Definitely:	There is clear evidence to suggest a causal relationship, and other possible contributing factors can be ruled out.
Probably:	There is evidence to suggest a causal relationship, and the influence of other factors is unlikely
Possibly	There is some evidence to suggest a causal relationship (e.g. the event occurred within a reasonable time after administration of the study procedure). However, the influence of other factors may have contributed to the event (e.g. the participant's clinical condition, other concomitant events).
Unlikely	There is little evidence to suggest there is a causal relationship (e.g. the event did not occur within a reasonable time after administration of the study procedure). There is another reasonable explanation for the event (e.g. the participant's clinical condition).
Not related	There is no evidence of any causal relationship.
Not Assessable	Unable to assess on information available.

16.2.3 Expectedness

Category	Definition
Expected	An adverse event which is consistent with the information about the procedure **clearly defined in this protocol.**
Unexpected	An adverse event which is not consistent with the information about the procedure **clearly defined in this protocol.**

* this includes listed events that are more frequently reported or more severe than previously reported

16.3 Recording adverse events

All adverse events will be recorded in the CRF with clinical symptoms and accompanied with a simple, brief description of the event, including dates as appropriate.

16.4 Procedures for recording and reporting Serious Adverse Events

All serious adverse events will be recorded in the medical records and the CRF, and the sponsor's AE log

All SAEs (except those specified in section 16.5 as not requiring reporting to the Sponsor) must be recorded on a serious adverse event (SAE) form. The CI/PI or designated individual will complete an SAE form and the form will be preferably emailed to the Sponsor within 5 working days of becoming aware of the event. The Chief or Principal Investigator will respond to any SAE queries raised by the sponsor as soon as possible.

Where the event is unexpected and thought to be related to the procedure this must be reported by the Investigator to the Health Research Authority within 15 days.

Completed forms for unexpected SAES must be sent within 5 working days of becoming aware of the event to the Sponsor<br>**Email forms to**<br>Research-incidents@ucl.ac.uk

Flow Chart for SAE reporting

Formatted: Numbering: Continuous

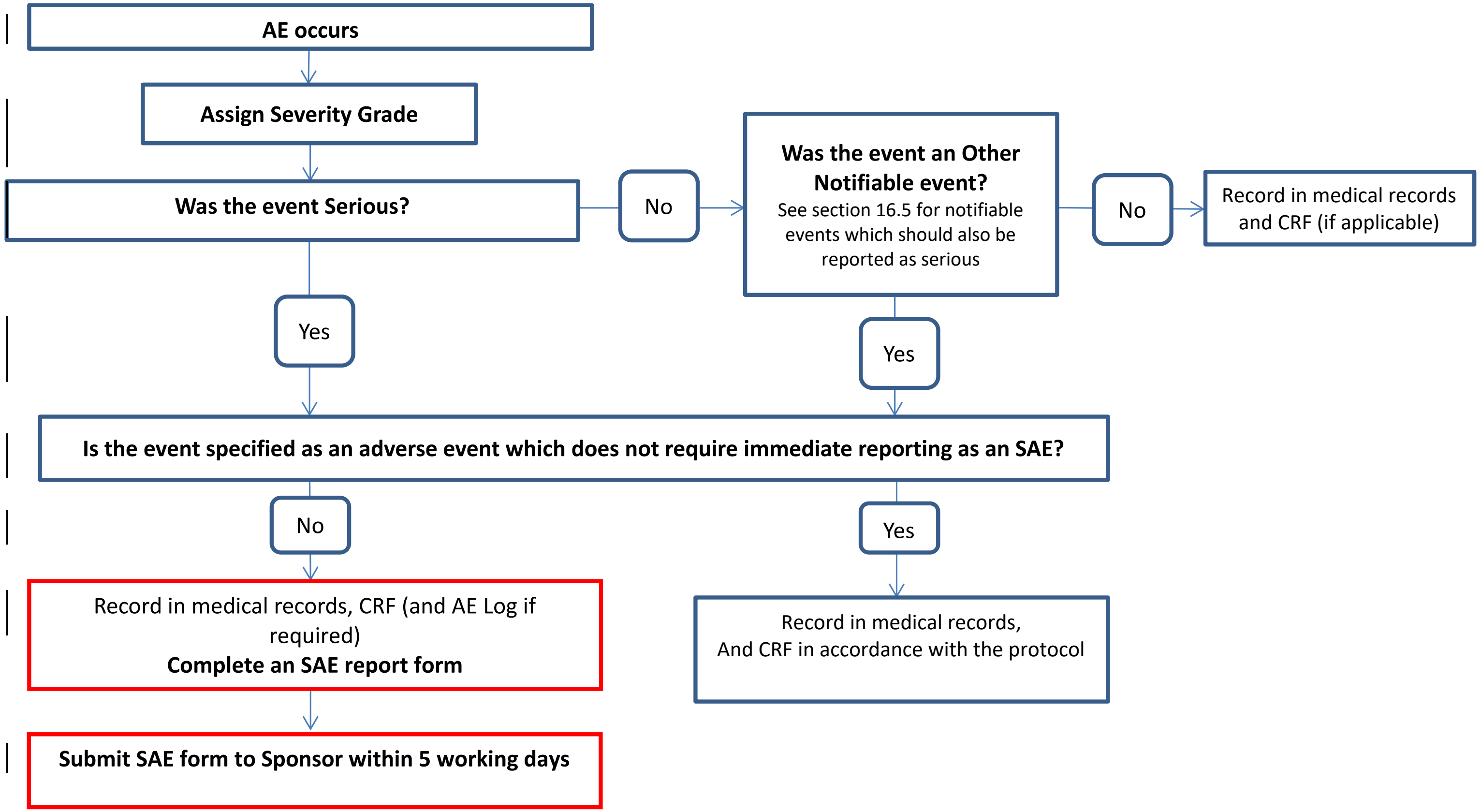

16.5 Serious Adverse Events that do not require reporting

Formatted: Numbering: Continuous

Some particular SAEs may not be reported to the sponsor, for example if they are expected to occur on a regular basis and offer no further new information to the safety profile or are related to the disease area of the participants. They will be reported if the frequency or severity of these events is unusual. These events will continue to be recorded in the CRF.

16.6 Reporting Urgent Safety Measures

If any urgent safety measures are taken the CI/ PI shall immediately and in any event no later than 3 days from the date the measures are taken, give written notice to the relevant REC and Sponsor of the measures taken and the circumstances giving rise to those measures.

16.7 Protocol deviations and notification of protocol violations

A deviation is usually an unintended departure from the expected conduct of the study protocol/SOPs, which does not need to be reported to the sponsor. The CI will monitor protocol deviations.

A protocol violation is a breach which is likely to effect to a significant degree –

(a) the safety or physical or mental integrity of the participants of the study; or
(b) the scientific value of the study.

The CI and sponsor will be notified immediately of any case where the above definition applies during the study conduct phase.

16.9 Trust incidents and near misses

An incident or near miss is any unintended or unexpected event that could have or did lead to harm, loss or damage that contains one or more of the following components:

a. It is an accident or other incident which results in injury or ill health.
b. It is contrary to specified or expected standard of patient care or service.
c. It places patients, staff members, visitors, contractors or members of the public at unnecessary risk.
d. It puts the Trust in an adverse position with potential loss of reputation.
e. It puts Trust property or assets in an adverse position or at risk.

Incidents and near misses will be reported to the Trust through DATIX as soon as the individual becomes aware of them.
A reportable incident is any unintended or unexpected event that could have or did lead to harm, loss or damage that contains one or more of the following components:

a) It is an accident or other incident which results in injury or ill health.
b) It is contrary to specified or expected standard of patient care or service.
c) It places patients, staff members, visitors, contractors or members of the public at unnecessary risk.
d) It puts the Trust in an adverse position with potential loss of reputation.
e) It puts Trust property or assets in an adverse position or at risk of loss or damage.

17 MONITORING AND AUDITING

The Chief Investigator will ensure there are adequate quality and number of monitoring activities conducted by the study team. This will include adherence to the protocol, procedures for consenting and ensure adequate data quality.

The Chief Investigator will inform the sponsor should he/she have concerns which have arisen from monitoring activities, and/or if there are problems with oversight/monitoring procedures.

18 TRAINING

The Chief Investigator will review and provide assurances of the training and experience of all staff working on this study. Appropriate training records will be maintained in the study files.

19 INTELLECTUAL PROPERTY

All intellectual property rights and know-how in the protocol and in the results arising directly from the study, but excluding all improvements thereto or clinical procedures developed or used by each participating site, shall belong to UCL. Each participating site agrees that by giving approval to conduct the study at its respective site, it is also agreeing to effectively assign all such intellectual property rights (“IPR”) to UCL and to disclose all such know-how to UCL with the understanding that they may use know-know gained during the study in clinical services and teaching to the extent that such use does not result in disclosure of UCL confidential information or infringement of UCL IPR.

20 INDEMNITY ARRANGEMENTS

University College London holds insurance against claims from participants for harm caused by their participation in this clinical study. Participants may be able to claim compensation if they can prove that UCL has been negligent. However, if this clinical study is being carried out in a hospital, the hospital continues to have a duty of care to the participant of the clinical study. University College London does not accept liability for any breach in the hospital’s duty of care, or any negligence on the part of hospital employees. This applies whether the hospital is an NHS Trust or otherwise.

21 ARCHIVING

UCL and each participating site recognise that there is an obligation to archive study-related documents at the end of the study (as such end is defined within this protocol). The Chief Investigator confirms that he/she will archive the study master file at UCL for the period stipulated in the protocol and in line with all relevant legal and statutory requirements. The Principal Investigator at each participating site agrees to archive his/her respective site’s study documents for 10 years and in line with all relevant legal and statutory requirements.

22 PUBLICATION AND DISSEMINATION POLICY

Study results will be published in peer-reviewed academic journals and also presented at public and scientific conferences. This work will also be highlighted on media channels, both traditional (TV, radio) and internet including UCL/ICN webpages and Twitter feeds.

23. Covid-19 safety precautions

This study will involve direct interaction between researchers and participants given the need for researchers to undertake some cognitive testing face to face. To minimize risk of CV19 infection, the following precautions will be taken.

- Researchers or participants will not be permitted to be involved if
  - They have active CV19 infection
  - They have been knowingly exposed to anyone with active CV19 infection
- Researchers and participants will use PPE (disposable gloves, face mask) where appropriate
- Wherever possible, social distancing will be observed

24. References

1. Ritchie K, Chan D (2021) *World Psychiatry 20, 52-53*
2. Mao L, Jin H, Wang M, et al. (2020) *JAMA Neurol* 77: 683-90.
3. Zhou H, Lu S, Chen J, et al. (2020) *J Psychiatr Res*. 129,98-102.
4. Rogers J, Chesney E, Oliver D et al. (2020) *Lancet Psychiatry* 7, 611-27
5. Helms J, Kremer S, Merdji H, et al. (2020) *N Engl J Med*, 382, 2268-70.
6. Almeria M, Cejudo J, Sotoca J, et al. (2020) *Brain Behav Immun Health* 9, 100163.
7. Needham E, Chou S, Coles A, et al. (2020) *Neurocrit Care* 32, 667-71.
8. Hampshire A, Trender W, Chamberlain S, et al. (2020) *medRxiv 2020.10.20.20215863*.
9. Woo M, Malsy J, Pöttgen J, et al. (2020) *Brain Comms* https://doi.org/10.1093/braincomms/fcaa205
10. Varatharaj A, Thomas N, Ellul M, et al. (2020) *Lancet Psychiatry* 7, 875-82.
11. Chen R, Wang K, Yu J, et al. (2021) *Front Neurol* Jan 20;11:573095. doi: 10.3389/fneur.2020.573095.
12. Meinhardt J, Radke J, Dittmayer C, et al. (2021) *Nat Neurosci* 24, 168-175.
13. Chan T, Marta M, Hawkins C, et al. (2020) *Curr HIV/AIDS Rep* 17, 514–521.
14. Christie S, Chan V, Mollayeva T, et al. (2018) *BMJ Open*. 2018;8(5):e015928.
15. Rogers J, Foord R, Stolwyk R, et al. (2018) *Neuropsychol Rev* 28, 285-309.
16. Merriman N, Sexton E, McCabe G, et al. (2019) *BMJ Open* 2019 Feb 27;9(2):e024429.
17. Cicerone K, Goldin Y, Ganci K, et al. (2019) *Arch Phys Med Rehab* 100, 1515-1533.
18. Clare L, Kudlicka A, Oyebode J, et al. (2019) *Health Technol Assessment* 23, 1-242.
19. DeLuca J, Chiaravalloti N, Sandroff B (2020) *Nat Rev Neurol* 16, 319-332.
20. Piras F, Borella E, Incoccia C, et al. (2011) *Eur J Phys Rehabil Med* 47, 149-75.
21. das Nair R, Cogger H, Worthington E, et al. (2016). *Cochrane Database Syst Rev* Sep Sept 1;9(9):CD002293.
22. das Nair R, Martin K, Lincoln N. (2016) Memory rehabilitation for people with multiple sclerosis. *Cochrane Database Syst Rev* Mar 23;3:CD008754.
23. Virk S, Williams T, Brunsdon R, et al. (2015) *Neurorehabilitation* 36, 367-77.
24. Grasso M, Broccoli M, Casillo P, et al. (2017) *Eur Neurol* 78, 111-117.
25. Pantoni L, Poggesi A, Diciotti S, et al. (2017) *J Alzheimers Dis* 60, 615-624.26.
26. Novakovic-Agopian T, Kornblith E, et al. (2018) J Neurotrauma 35, 2784-2795.
27. Fenwick E, O'Brien B., Briggs A. (2004) *Health Econ* 13, 405-415.
28. National Institute for Health and Clinical Excellence (2013). Guide to the methods of technology appraisal. London: National Institute for Health and Clinical Excellence.

25. APPENDICES

Include here supplementary information and documents that will support the protocol and information contained therein, e.g. PIS, ICF, schedule visit, assessment tools, delegation log, case report forms, questionnaires, scales, tables, charts, diagrams, manufacturer's brochures.

STATISTICAL ANALYSIS PLAN

FOR

CICERO WS2 RCT

Date: 05/09/2024

Version: 1

North Wales Organisation for Randomised Trials in Health (NWORTH)
Bangor University, College of Health & Behavioural Sciences,
Institute of Medical & Social Care Research, Y Wern, Normal Site, Holyhead Road, Bangor, Gwynedd LL57 2PZ
Telephone: 01248 388095 Email: nworth@bangor.ac.uk Website: http://nworth-ctu.bangor.ac.uk/

Cognitive Impairment in Long Covid: PhEnotyping and RehabilitatiOn (CICERO)

Trial registration number: IRAS Project ID: 302920

Based on protocol version: 143067 CICERO Protocol v1.11 1May2024

Chief Investigator: Professor Dennis Chan, Institute of Cognitive Neuroscience, UCL

Co-Investigators:
Dr Aida Suarez Gonzalez, Dementia Research Centre, UCL
Dr Zoe Hoare, University of Bangor
Dr Nathan Bray, University of Bangor
Dr Jason Lim (public-patient initiative lead)
Professor Chris Kipps, University Hospital Southampton NHS
Dr Ross Dunne, Greater Manchester Mental Health NHS Trust

Project Manager: Gina Gilpin

Trial Statistician: Rachel Evans

Rachel Evans<br>Author's Name	Date: 05/09/2024<br>Signature
Dr. Zoë Hoare<br>Second Statistical Advisor	Date: 05/09/2024<br>Signature
Prof. Dennis Chan<br><br>Chief Investigator	Date:<br>5/9/24<br>Signature

DOCUMENT HISTORY

Updated version no.	Effective date	Authorship	Section changed	Summary of changes
1	05/09/2024	R Evans	N/A	N/A

TABLE OF CONTENTS

1. Acronyms and definition of terms 4
2. Statistical Analysis Plan Authorship 5
3. Introduction 5
   - 3.1 Background and Rationale 5
   - 3.2 Trial Aims and Objectives 5
   - 3.3 Trial Design 6
   - 3.4 Participant Population 6
   - 3.5 Participant Flow Diagram 8
4. Statistical Principles 9
   - 4.1 Sample size justification 9
   - 4.2 Randomisation 9
   - 4.3 Levels of confidence and p-values 9
   - 4.4 compliance/adherence 9
   - 4.5 Protocol Violations 10
   - 4.6 Missing Data 10
   - 4.7 Assumption Checking 11
5. Data 12
   - 5.1 Data Collection and Handling 12
   - 5.2 Time Points of Outcomes Measures 12
   - 5.3 Definitions and calculations of outcome measures 14
   - 5.4 Safety data 14
6. Statistical analyses 14

6.1 Analysis Time Frame 14
6.2 Baseline Analysis 14
6.3 Interim Analysis 14
6.4 Participant Flow and CONSORT Reporting 14
6.5 Descriptive statistics 16
6.6 Analysis of primary outcome 16
6.7 Analysis of secondary outcomes 16
6.8 Subgroup analyses 17
6.9 Sensitivity analyses or model testing 17
6.10 Exploratory analyses 18
7. Process Evaluation 18
8. Software 18
9. References 18
10. Appendices 19

1. ACRONYMS AND DEFINITION OF TERMS

Acronym	Meaning
AE	Adverse Event
BSGI	Bangor Goal Setting Interview
CONSORT	Consolidated Standards for Reporting Trials
CV19	Covid-19
CTU	Clinical Trials Unit
DSH	Data Safe Haven
ITT	Intention to Treat
MAR	Missing at Random
MCAR	Missing Completely at Random
MI	Multiple imputation
MRI	Magnetic Reasoning Imaging
NHS	National Health Service
NWORTH	North Wales Organisation for Randomised Trials in Health
PI	Principal Investigator
RCT	Randomised Controlled Trial
SAE	Serious Adverse Event

SAP	Statistical Analysis Plan
UCL	University College London
WS1	Work Stream 1
WS2	Work Stream 2

2. STATISTICAL ANALYSIS PLAN AUTHORSHIP

The Statistical analysis plan (SAP) has been authored by Rachel Evans, Senior Statistician with input from Dr Zoë Hoare (Principal Statistician), Gina Gilpin (Project Manager), Aysha Patel (Research Assistant), Dr Aida Suarez Gonzalez (Co-applicant) and Dr Denis Chan (Chief Investigator).

The quantitative Statistical analysis described in this document relates to WS2 of the trial (RCT) and will be completed by Rachel Evans at NWORTH CTU with oversight from Zoë Hoare. Analysis of WS1 will be reported separately. Analysis will be conducted blinded, and an initial blinded results report will be produced and sent to the team. Once all blinded analysis listed in the SAP has been conducted the statisticians will be officially unblinded following NWORTHs SOPs, and further analysis on intervention data, listed in section 6.5, 6.8 and 6.9 will be conducted.

3. INTRODUCTION

3.1 BACKGROUND AND RATIONALE

Cognitive impairment is recognised as a major component of long Covid, present in 25-75% of affected individuals, but little is known about the nature of this impairment, or how it can be treated. The associated loss of functional ability has major consequences for affected people, their families and the wider economy given the problems caused in terms of return to work. The aim of this study is to determine the nature of the cognitive impairment and deliver cognitive rehabilitation to help a return to normal life.

3.2 TRIAL AIMS AND OBJECTIVES

Workstream 2 will develop and test the effectiveness of a neuropsychological rehabilitation intervention to support recovery from cognitive Covid.

Aims:

- Can a neuropsychological rehabilitation programme (COVID Rehab) improve the cognitive and functional outcomes of people with cognitive Covid?

- What is the incremental cost-effectiveness of COVID-Rehab compared to standard clinical care for cognitive Covid?*[1]
- What is the cost per quality-adjusted life year (QALY) of COVID Rehab compared to standard clinical care for cognitive Covid, and does this fall below the NICE threshold of £20,000 to £30,000 per QALY?*

Objectives:

- To develop and test the effectiveness of a neuropsychological rehabilitation intervention programme for cognitive Covid (COVID-Rehab) to deliver improvements in clinically relevant outcomes including quality of life and functional ability.

- To investigate the incremental cost-effectiveness of the CIVIC-Rehab intervention compared to standard clinical care, using multiple measures of effect.*

3.3 TRIAL DESIGN

WS2 – The second workstream will develop a suite of phenotype-specific and evidence-based neuropsychological rehabilitation packages targeted at the cognitive domains primarily affected in cognitive Covid, using functional and quality of life outcomes to measure treatment efficacy. This collection of strategies will be informed by outcomes from workstream 1 and co-produced in collaboration with a group of people living with cognitive Covid-19 (we will refer to this group as Experts by Experience). This will be a parallel individually randomised controlled trial, comparing the study intervention (cognitive rehabilitation) with the clinical standard of care (current clinical management pathways for long Covid, including treatment for fatigue, respiratory disorders, sleep, anxiety and depression).

3.4 PARTICIPANT POPULATION

Participants will be recruited from the cohorts of patients with long Covid and cognitive impairment seen in the Cognitive Disorders Clinics held at University Hospitals Sussex

---

[1] *Health economic analysis will be conducted outside of NWORTH, the current SAP and associated report will not cover analyses associated with these aims and objectives.

NHS Trust (PI Chan), Southampton NHS Trust (PI Kipps), and Greater Manchester Mental Health Trust (PI Dunne). They will initially be approached by the local PIs or associated researchers working at each study site and informed consent for participation will be obtained.

Inclusion Criteria:

- Aged between 30 and 60 years
- Evidence of prior CV19 infection
  - *either* positive CV19 PCR
  - *or* positive CV19 antibody test
  - *or* acute symptoms consistent with the recognised core features of acute CV19 infection and post-acute symptoms consistent with the recognised core features of long Covid

  Cognitive impairment persisting more than three months after the acute CV19 infection, defined in terms of subjective reports of cognitive decline post-infection.

Exclusion Criteria:

- Cognitive impairment prior to CV19 infection
- Occurrence of acute neurological disorder, such as stroke or encephalitis, that could give rise to cognitive sequelae.
- People who are on any medications that are considered by the study investigators to have significant adverse effects on cognition.
- A pre-existing major psychiatric or medical disorder that is considered by the study investigators to have potential to affect cognition.
- High alcohol intake
- Recreational drug use
- Loss of mental capacity such that the affected individual is unable to give informed consent.
- Participants will not be eligible for Workstream 2 if they do not exhibit significant impairment on baseline cognitive assessments, defined as impairment in two or more cognitive domains, as they will not gain from cognitive rehabilitation (i.e. for inclusion Scores >= 1 SD below age-adjusted mean in at least two of the following cognitive domains: immediate memory, visuospatial memory, language, attention, delayed memory, information-processing speed, executive functioning, inhibition, and verbal fluency)

- Participants with pacemakers or other implanted devices, those with metal foreign bodies (e.g. shrapnel from war injuries) and those who have had certain types of surgery will be excluded from the MRI substudy. Although MRI is not known to affect the unborn child, we will also exclude subjects who may be pregnant just to be on the safe side.

3.5 PARTICIPANT FLOW DIAGRAM

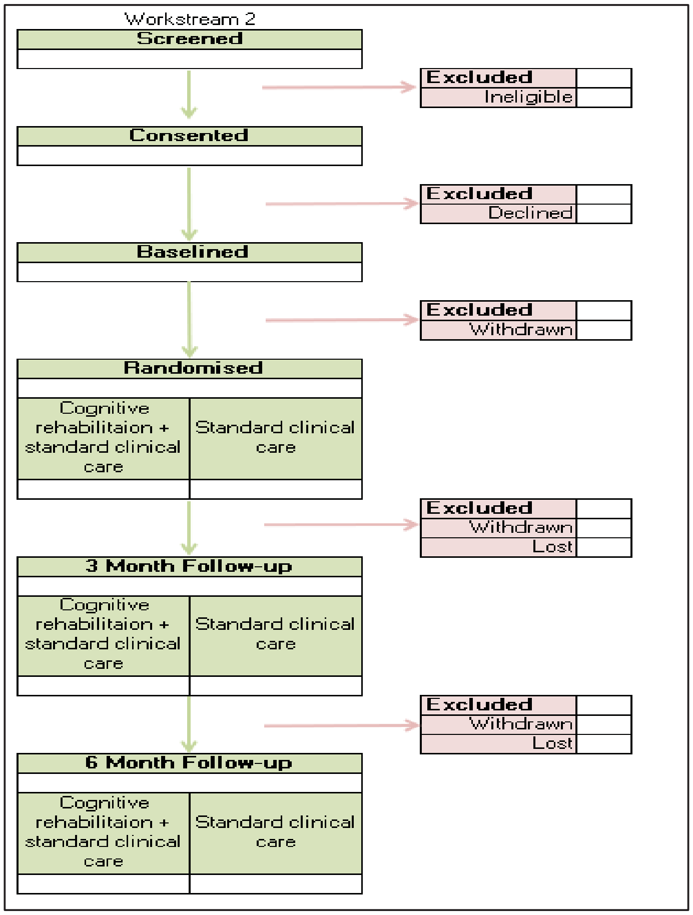

Figure 1: CICERO WS2 Participant Flow Diagram

4. STATISTICAL PRINCIPLES

All those randomised will be included in the analysis based on an intention to treat (ITT) principal.

4.1 SAMPLE SIZE JUSTIFICATION

A sample of 88 (44:44) will be required to detect an effect of 0.7 on the BGSI at 3 months with 90% power at a 5% significance level. Incorporating an attrition of 25% will require recruitment and randomisation of 118 participants (59:59). Therefore, 120 participants will be recruited from the same clinics. Participants in WS1 can if desired take part in WS2.

4.2 RANDOMISATION

Randomisation will take place once consent and baseline data collection has been completed. Randomisation will be on a 1:1 allocation (intervention:control) via a secure online system hosted by NWORTH, Bangor University. The system will employ a dynamic adaptive randomisation algorithm. The randomisation will be stratified for centre (London/Sussex/Southampton/Manchester). A member of the research team at UCL will enter the participants details into the web form and perform the randomisation, which will either display directly on screen, if the researcher is unblinded or notification of the randomisation result will be sent to the designated parties to organise ongoing contact.

4.3 LEVELS OF CONFIDENCE AND P-VALUES

All statistical tests will be two-sided and performed using 5% level of significance. All confidence intervals presented will be 95%, unless stated otherwise.

Secondary outcomes will not be adjusted for multiple comparisons, as standard. The primary outcome is powered for at the significance level stated. Analysis of all secondary outcomes is considered exploratory, and any statements of results related to these outcomes will be reported appropriately.

4.4 COMPLIANCE/ADHERENCE

There is no planned analysis for compliance and treatment adherence. Sensitivity analysis will be run on the number of intervention sessions received, see section 6.9 for further details.

4.5 PROTOCOL VIOLATIONS

Definition of protocol violations and deviations. Violation is an intended failure to adhere to the protocol such as wrong treatment being prescribed or administered or incorrect data being collected and documented. A protocol deviation is an unintended failure to adhere to the protocol and examples include errors in applying inclusion/exclusion criteria or missed follow-up visits due to error. Protocol deviations and violations will be monitored and detailed descriptively in the analysis report. If protocol deviation occurrences rise above 10% then sensitivity analysis as detailed in section 6.5 will be conducted. Some examples of protocol deviations which might occur in this study include;

- Patients not receiving randomised allocation.
- Ineligible participants being randomised.
- Participants receiving their BGSI goals after completing it at baseline.

ITT is the expected standard for RCTs and demonstrates the effectiveness of the intervention in the pragmatic context. Per protocol will provide evidence of efficacy and if there is a large difference then there may be a difficulty in implementation of the intervention which needs fixing before the intervention would be adoptable by the NHS.

4.6 MISSING DATA

Every effort will be taken to minimise missing data. If imputation (e.g., mean substitution) is part of the validated measure it will be performed, see Appendix 1 for measure scoring rules. Where there are no missing data rules for the measure, if the number of missing items on an outcome is 20% or less, then the missing value for the item will be substituted by the individual's mean score for the remaining items on the scale (Bono, Ried, Kimberlin and Vogel 2007). If there are more than 20% missing items in the scale the outcome measure will not be calculated for the participant at that time point and multiple imputation methods will be used.

In accordance with the recommendations of Jakobsen, Gludd, Wetterslev and Winkel (2017), if the proportion of missing data is less than 5%, no imputation of missing data will be performed, and analyses will be based on complete cases. If the proportion of missing data is equal to or greater than 5%, a missing completely at random test devised by Little (1998) will be performed to assess whether the data is missing completely at random (MCAR). If this test indicates that data is missing completely at

random then analyses will be based on complete cases, else independent t-tests and Chi-square tests will be conducted to investigate whether the data is missing at random (MAR). If these tests suggest that the missing data is MAR, then predictive mean matching multiple imputation methods will be employed. Otherwise, additional modelling guided by clinical knowledge would be required to simulate the missing data mechanism and impute the missing data.

For multiple imputations, the number of imputations completed will be dependent upon the percentage of missing data (White et al., 2011). The missing outcome measures will be imputed using Allocation Group and Stratification factors (Site) and other variables that are deemed to be predictors of missingness. Variables will be assessed for being predictors of missingness by running statistical tests on completers versus non completers and evaluating if any differences are present between the two.

Variables to be assessed as predictors of missingness include;

- Age
- Sex
- Ethnicity
- Education
- Disability
- Occupation
- Drugs
- CV19 (diagnosed conditions (before/during)
- Number of vaccines
- Cognition

If MI is used, complete case analysis will also be conducted as a sensitivity analysis to check the assumptions of the MI models, see section 6.8. In line with the recommendations of Jakobsen et al (2017), if more than 40% of data is missing for an outcome measure the data will not be imputed and an analysis will be conducted on complete cases, though the results of the analysis will be interpreted tentatively.

4.7 ASSUMPTION CHECKING

All assumptions relating to the models will be checked and evaluated whether appropriate to use with the data. If any of the assumptions are substantially violated, then appropriate non-parametric tests will be conducted.

During data cleaning, the Trial Statistician will assess the data for outliers by running Grubbs test (Grubbs 1969) and visually inspecting a boxplot. Any outliers identified will

be queried to identify whether the outlier is a data entry error, a measurement error or to confirm that it is a genuinely unusual value. Once this has been clarified the data will be amended if necessary or will remain unchanged if identified to be correct. No outliers will be discarded from analysis if they are within range a.

The distribution of the continuous data will be checked and depending on the result of these checks a decision will need to be made as to whether a transformation should be applied to the data and if so, which transformation should be used. If a transformation is required, the distribution of the transformed data will be checked. Analysis will be reported on the original scale, transforming data back. If a transformation is inappropriate/unhelpful then nonparametric analysis methods will be considered.

For the logistic regressions, if detection rates (number of responses observed) are very low, where appropriate, either a Firth's Bias-Reduced Logistic Regression will be conducted, or descriptive statistics reported.

5. DATA

5.1 DATA COLLECTION AND HANDLING

Data collection and entry onto a REDCap database, which will save data directly to UCL's data safe haven (DSH), will be undertaken at the individual sites. Cognition data will be collected via an excel spreadsheet (behavioural database) and stored on the DSH. Cleaning and analysis will be undertaken by NWORTH using standard, secure, anonymous procedures for handling participant data. For further details, please refer to the Data Management Plan.

5.2 TIME POINTS OF OUTCOMES MEASURES

Outcome measures are collected at baseline, 3 months and 6 months post-randomisation. Appendix 1 contains a list of all data collected during the study.

The primary outcome is participant-reported goal attainment at 3 months post-randomisation. This will also be collected as a secondary outcome at the 6 month post-randomisation follow up. This will be collected using the Bangor Goal Setting Interview (BGSI).

Secondary outcomes will be:

- Goal Satisfaction Scale ((using the Bangor Goal Setting Interview Scale)
- Cognitive function (tested using the cognitive batteries employed for Workstream 1)
- Quality of life (EQ-5D-5L)*
- Life Space Assessment
- Social Functioning (SF-DEM)
- Instrumental Activities of Daily Living (IADL) Scale
- Generalised Anxiety Disorder Assessment (GAD-7)
- Patient Health Questionnaire (PHQ-8)
- Chalder Fatigue Scale
- Pittsburgh Sleep Quality (PSQI)
- DePaul Symptom Questionnaire – Post-Exertional Malaise (DSQ-PEM).
- Client Service Receipt Inventory (CSRI)*

Cognitive function will be assessed using three complementary assessments (expanded below), to maximise understanding of cognition

- Cognitive function assessment 1 – battery of Cognitive tests
    - The Repeatable Battery for the Assessment of Neuropsychological Tests (RBANS)
    - The Delis-Kaplan Executive Function System: The Trail Making Test (Condition 2: Number Sequencing & Condition 4: Number-Letter Switching)
    - The Delis-Kaplan Executive Function System: Colour-Word Interference
    - The Delis-Kaplan Executive Function System: Phonemic Fluency
    - Wechsler Adult Intelligence Scale III: Digit Span
    - Test of Premorbid Functioning (TOPF)
- Cognitive function assessment 2 – online battery of cognitive tests
    - Gorilla
    - 4 Mountains (4MT)
- Cognitive function assessment 3
    - Neotiv suite

*Health economic data not being analysed at by NWORTH.

5.3 DEFINITIONS AND CALCULATIONS OF OUTCOME MEASURES

For a full definition and respective calculations of each outcome measure please see Appendix 2.

5.4 SAFETY DATA

The number (and proportion) of participants experiencing AE/SAEs will be reported. No formal statistical testing will be undertaken of the safety data.

6. STATISTICAL ANALYSES

6.1 ANALYSIS TIME FRAME

Table 1: Summary of expected timelines for analysis

TASK	EXPECTED DATE
Data Cleaning	August 2024
Data locking*	August 2024
Analysis completed**	September 2024

**The completion of data lock is dependent on the date of the final follow up, data entry from all sites, responses to data queries and sign off of the statistical analysis plan.*

***a minimum of 1 month between data lock and analysis report delivery is required.*

6.2 BASELINE ANALYSIS

A separate baseline analysis will not be conducted. A section of the main analysis and report will detail the characteristics of the study sample at baseline. Descriptive statistics will be used to describe the sample, see section 6.5.

6.3 INTERIM ANALYSIS

No interim analysis is planned.

6.4 PARTICIPANT FLOW AND CONSORT REPORTING

The patient flow information, as shown in Figure 2, as advised by CONSORT reporting standards, will be completed with values relating to participants numbers. Data will be presented on screening, eligibility, recruitment, treatment discontinuation and withdrawn/lost to follow up. Where possible reasons for ineligibility or non-recruitment will be reported. From these data related eligibility, recruitment and retention rates will be calculated and presented in the final analysis report.

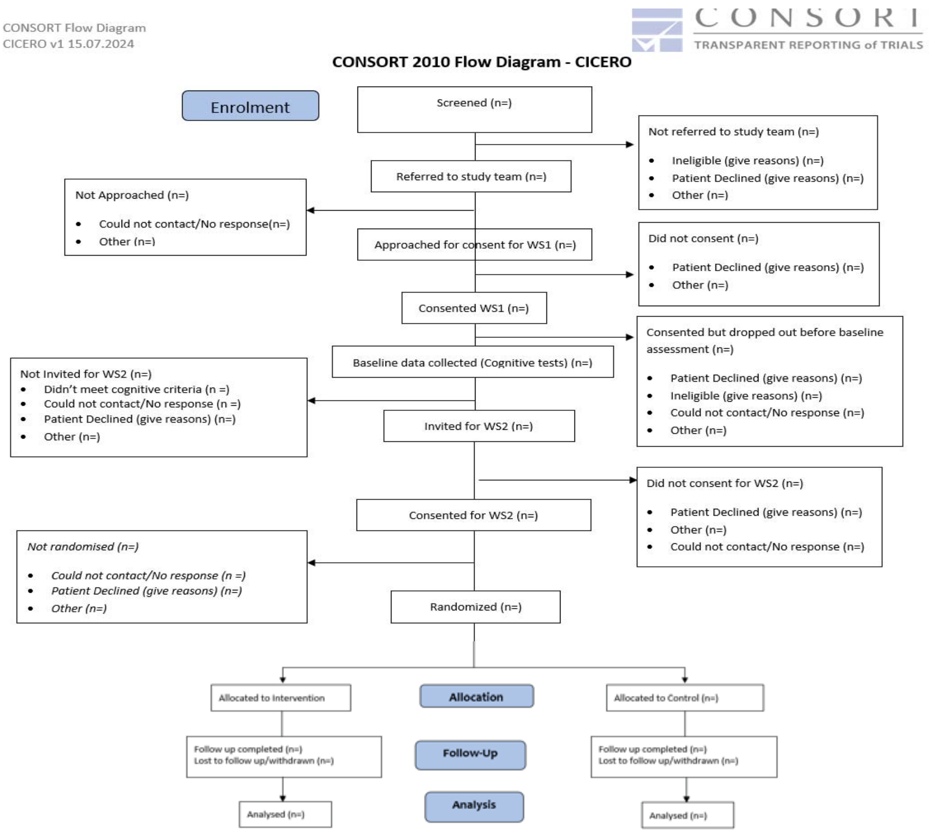

Figure 2: Participant Flow diagram to be completed in line with CONSORT reporting guidelines.

6.5 DESCRIPTIVE STATISTICS

Descriptive statistics of the data will be presented. This will include randomisation figures, demographics, COVID19 data and outcome measure data along with completeness levels to assess missing data.

For all descriptive statistics continuous measures will be reported with mean values and standard deviations and categorical variables presented with counts and related percentages. If data are not normally distributed, then medians and interquartile ranges will be reported. Categorical variables will be reported with counts and related percentages. The following data will be presented:

- Participant demographics and sample characteristics
- Outcome measures at each timepoint (listed in section 5.2)
- Cognitive Assessments of cognitive function (listed in section 5.2)
- *Intervention data summary; number of sessions attended.

**Presented in unblind report once all primary analysis listed in this SAP is complete.*

All data will be presented overall and split by allocation group.

6.6 ANALYSIS OF PRIMARY OUTCOME

The primary outcome, the BGSI (attainment score) at the 3 months, will be analysed using a multi-level (mixed effect) linear regression model to assess the differences between the two treatment arms. Stratification variables (Site) will be incorporated into the model as a random effect along with gender, age (continuous), years of education (continuous) and RBANS composite score at baseline as fixed factors. Baseline BGSI attainment score will also be incorporated to account for any differences at baseline.

The BGSI at 6 months will be analysed as a secondary outcome using the same model structure as the primary. Unadjusted (section 6.5) and adjusted results will be presented with associated 95% confidence intervals.

6.7 ANALYSIS OF SECONDARY OUTCOMES

For all secondary outcomes the continuous variables will be analysed using multi-level (mixed effect) linear regression models and categorical (binary) measures will be analysed using multi-level (mixed) logistic regression models. The secondary models will follow the same structure as the primary, including Stratification variables (Site) as

a random effect along with gender, age (continuous), years of education (continuous) and RBANS composite score at baseline as fixed factors and baseline score of the outcome to account for baseline differences. Unadjusted (section 6.5) and adjusted results will be presented with associated 95% confidence intervals.

Appendix 1 details all outcomes for the trial, variable types and their corresponding analysis.

6.8 SUBGROUP ANALYSES

Subgroup analysis of “responders” and “non-responders” will be run by presenting demographic data and baseline scores for the two groups (responder vs non-responders) to explore if response can be predicted.

“Responders” will be defined as scoring at least 2 points difference from baseline in goal attainment rating measured on the BGSI (primary outcome) at each timepoint respectively.

This will also be carried out for those that significantly surpass the 2-point improvement defined as those who achieve 4 or more points difference at follow up compared to baseline.

6.9 SENSITIVITY ANALYSES OR MODEL TESTING

If MI is used, then complete case analysis will be run to evaluate any impacts that MI has had on the results. This will be done on the primary outcome (BGSI attainment at 3 months) and any secondary outcomes that are statistically significant with MI analysis.

A sensitivity analysis will be run for the number of intervention sessions attended*; this will be run by including number of sessions in the analysis model (as a continuous variable) along with the interaction with group.

An additional sensitivity analysis will be conducted on the primary outcome (BGSI attainment at 3 months) to assess impact of intervention treatment windows*. Along with including number of sessions in the model as above, participants who did not receive the intervention within the correct window for any one session will be excluded (not including missed sessions).

**Presented in unblind report once all primary analysis listed in this SAP is complete.*

Sensitivity analysis will be conducted on the primary outcome (BGSI attainment score) at 2 and 6 months only including the participants whose data was collected within the aimed timeframe, that is within 2 weeks of the follow up due date.

6.10 EXPLORATORY ANALYSES

Exploratory analysis will be conducted to evaluate the impacts that BGSI - Readiness, importance and difficulty scores have on attainment scores. The BGSI attainment analysis model will be re-run at 3 months and 6 months including these variables in the models.

7. PROCESS EVALUATION

There is no process evaluation.

8. SOFTWARE

All quantitative analysis will be completed using Stata v18 or later.

9. REFERENCES

Bono, C.A., Ried, L.D., Kimberlin, C.A., & Vogel, W.B. (2007). Missing data on the Center for Epidemiological Studies Depression Scale: A comparison of four imputation techniques. Research in Social & Administrative Pharmacy, 3, 1-27.

Grubbs, F. E. (1969). Procedures for detecting outlying observations in samples. Technometrics, 11(1), 1-21.

Little, R. J. A. (1988). A Test of Missing Completely at Random for Multivariate Data with Missing Values. Journal of the American Statistical Association, 83(404), 1198–1202. https://doi.org/10.1080/01621459.1988.10478722

Jakobsen, J. C., Gluud, C., Wetterslev, J., & Winkel, P. (2017). When and how should multiple imputation be used for handling missing data in randomised clinical trials–a practical guide with flowcharts. BMC medical research methodology, 17(1), 162.

White, I. R., Royston, P., & Wood, A. M. (2011). Multiple imputation using chained equations: issues and guidance for practice. Statistics in medicine, 30(4), 377-399.

10. APPENDICES

Appendix 1 – Table of data collected for CICERO WS2 and associated analysis.

Measures to be collected (as stated in the protocol)	Variable/Data Type	Analysis	Timepoints for analysis
Demographics	n/a	Descriptive statistics only, some may be used in model as covariates	n/a
CV19 symptoms	n/a	Descriptive statistics only, some may be used in model as covariates	n/a
CV19 vaccination	n/a	Descriptive statistics only, some may be used in model as covariates	n/a
Bangor Goal Setting Interview (BGSI) (attainment/performance score)	Continuous	Multi-level linear regression model	*3 and 6 months
Bangor Goal Setting Interview (BGSI) (satisfaction)	Continuous	Multi-level linear regression model	3 and 6 months
Bangor Goal Setting Interview (BGSI) (Readiness, importance and difficulty)	n/a	Descriptive statistics only, some may be used in model as covariates	n/a
Quality of life (EQ-5D-5L)	Not applicable – health economic data		
Life Space Assessment (LSQ)	Continuous	Multi-level (mixed) linear regression model	3 and 6 months
Social Functioning (SF-DEM)	Continuous	Multi-level (mixed) linear regression model	3 and 6 months

Measures to be collected (as stated in the protocol)	Variable/Data Type	Analysis	Timepoints for analysis
Instrumental Activities of Daily Living (IADL) Scale	Continuous	Multi-level (mixed) linear regression model	3 and 6 months
Generalised Anxiety Disorder Assessment (GAD-7)	Continuous	Multi-level (mixed) linear regression model	3 and 6 months
Patient Health Questionnaire (PHQ-8)	Continuous	Multi-level (mixed) linear regression model	3 and 6 months
Chalder Fatigue Scale	Continuous	Multi-level (mixed) linear regression model	3 and 6 months
Pittsburgh Sleep Quality (PSQI)	continuous	Multi-level (mixed) linear regression model	3 and 6 months
DePaul Symptom Questionnaire – Post-Exertional Malaise (DSQ-PEM).	binary	Multi-level logistic regression model	3 and 6 months
CSRI	Not applicable – health economic data		
Cognitive function assessment 1 – battery of Cognitive tests<br>• RBANs,<br>• Delis-Kaplan (trail making, number-letter switching, colour-word interference, phonemic fluency),<br>• Wechsler,<br>• TOPF	Several	TBC	3 and 6 months
Cognitive function assessment 2 – online battery of cognitive tests	Several	TBC	3 and 6 months

Measures to be collected (as stated in the protocol)	Variable/Data Type	Analysis	Timepoints for analysis
• Gorilla<br>• 4 Mountains (4MT)			
Cognitive function assessment 3<br>• Neotiv suite	Several	TBC	3 and 6 months

*Primary outcome BGSI at 3-month endpoint

Appendix 2 – Scoring information for validated outcomes.

DEFINITION	ITEM CODING	SCORING	SUBSCALES	MISSING VALUE RULES	THRESHOLDS	INTERPRETATION (DIRECTION)
**Modified Life Space Questionnaire**<br>**Ref**: Stalvey, b. T., owsley, c., sloane, m. E., & ball, k. (1999). The life space questionnaire: a measure of the extent of mobility of older adults. Journal of applied gerontology, 18(4), 460-478.<br>**Scoring**: above reference page 464						
Assesses a person's life space and level of dependence when moving within a Certain life space	9-item measure **(**(Yes – 1 or No - 0*)<br><br>*recode required from database.	The overall score is calculated by summing the scores. The total score ranges from 0 to 18.<br><br>*CICERO study has omitted one item which was not relevant, hence scale is 'modified', total score for CICERO ranges from 0 to 16	None found	None.	None.	Larger scores signify a larger life space.
**Social Functioning (SF-DEM)**<br>**Ref**: Sommerlad, a., singleton, d., jones, r., banerjee, s., & livingston, g. (2017). Development of an instrument to assess social functioning in dementia: the social functioning in dementia scale (sf-dem). Alzheimer's & dementia: diagnosis, assessment & disease monitoring, 7, 88-98<br>**Scoring**: https://www.ucl.ac.uk/psychiatry/sites/psychiatry/files/sf-dem_instrument.pdf						
A 20-item questionnaire to assess social functioning in dementia.	Items 1-11 are scored 0 (Never) to 3 (Very often). 12-17 are reverse coded.<br><br>The final 3 items are unscored summary questions.	Each section (1-3) is summed. The final score is the total sum of sections 1 to 3 (i.e., questions 1 to 17)<br><br>A total score therefore ranges from 0 to 51.	3 sections:<br>• Spending time with other people (1-7)<br>• communicating with other people (8 -13)<br>• Relationships (14-17)	None.	None.	A high score indicates a better social function.
**Instrumental Activities of Daily Living (IADL) Scale**<br>**Ref**: Lawton, m. P., & brody, e. M. (1969). Assessment of older people: self-maintaining and instrumental activities of daily living. The gerontologist, 9(3_part_1), 179-186.<br>**Scoring**: https://nursing.ceconnection.com/ovidfiles/00000446-200804000-00023.pdf						

An 8-item scale to assess independent living skills	For each category, a score of 0 or 1 will be scored depending on the client's highest functional level.<br><br>(*Requires a recode from database*)	The final score is the total of each category. Scores therefore range from 0 - 8	None.	None.	None.	A higher score indicates higher function and independency.
**Generalised Anxiety Disorder Assessment (GAD-7)**<br>**Ref**: spitzer, r. L., kroenke, k., williams, j. B., & löwe, b. (2006). A brief measure for assessing generalized anxiety disorder: the GAD-7. Archives of internal medicine, 166(10), 1092-1097.<br>**Scoring**: https://jamanetwork.com/journals/jamainternalmedicine/fullarticle/410326						
A 7-item questionnaire to assess for generalized anxiety disorder.	Items scored on a 4-point Likert scale from 0 (Not at all) to 3 (Nearly every day).	Item scores are summed together to calculate the total score ranging from 0 to 21.	None.	None.	0–4: minimal<br>5–9: mild<br>10–14: moderate<br>15–21: severe	Higher scores indicate higher anxiety severity<br><br>i.e. higher scores worse
**Patient Health Questionnaire (PHQ-8)**<br>**Ref**: kroenke, k., strine, t. W., spitzer, r. L., williams, j. B., berry, j. T., & mokdad, a. H. (2009). The PHQ-8 as a measure of current depression in the general population. Journal of affective disorders, 114(1-3), 163-173.<br>**Scoring**: https://www.psychologywizard.net/uploads/2/6/6/4/26640833/kroenke_phq8.pdf						
An 8-item questionnaire to assess for depression.	Each question is scored 0 (Not at all) to 3 (Nearly every day).	The final score is the total of each question.	None.	If more than 1 item is missing, set the value of the scale to missing.		A higher score indicates greater depression.
**Chalder Fatigue Scale (CFQ 14)**<br>**Ref**: Chalder, T., Berelowitz, G., Pawlikowska, T., Watts, L., Wessely, S., Wright, D., & Wallace, E. P. (1993). Development of a fatigue scale. Journal of psychosomatic research, 37(2), 147-153<br>**Scoring**: **HTTPS://ME-PEDIA.ORG/WIKI/CHALDER_FATIGUE_SCALE** and https://www.goodmedicine.org.uk/files/assessment,%20chalder%20fatigue%20scale.pdf						

<table>
<tr>
<td>An 14-item questionnaire to assess the extent and severity of fatigue.</td>
<td>4-point scale ranging from the asymptomatic to maximum symptomology, such as ‘Better than usual’, ‘No
worse than usual’, ‘Worse than usual’ and ‘Much worse
than usual’.

Two scoring systems bimodal counts the number of symptoms, the other weights and intensity of the symptoms:
<table>
<tr><th colspan="3">Scoring schemes for the Chalder Fatigue Scale</th></tr>
<tr><th></th><th>Bimodal score</th><th>Likert score</th></tr>
<tr><td>Less than usual</td><td>0</td><td>0</td></tr>
<tr><td>No more than usual</td><td>0</td><td>1</td></tr>
</table>
</td>
<td>For both scoring systems (bimodally and Likert) total score is calculated by summing items.

Total scores range from:
0 – 14 (bimodal)
0 – 42 (Likert)

CICERO used the Likert version of the scale therefore the measure will be scored in line wit this.</td>
<td>2 subscales:
• Physical fatigue (q1-8)
• psychological fatigue (q9-14)</td>
<td>None.</td>
<td>global binary fatigue score of 3 or less represents scores of those who are not fatigued, with scores of 4 or more equating to ‘severe fatigue’</td>
<td>Higher scores indicate higher extent and severity of fatigue.</td>
</tr>
</table>

	More than usual 1 2<br><br>Much more than usual 1 3					
**Pittsburgh Sleep Quality Index (PSQI)**<br>**Ref**: buysse, d. J., reynolds iii, c. F., monk, t. H., berman, s. R., & kupfer, d. J. (1989). The pittsburgh sleep quality index: a new instrument for psychiatric practice and research. Psychiatry research, 28(2), 193-213.<br>**Scoring: HTTPS://BIOLINCC.NHLBI.NIH.GOV/MEDIA/SEARCH?Q=PSQI**						
A 24-item questionnaire, with 19 self-rated questions and 5 rated by bed partner, to assess overall sleep quality.	The 19 self-rated questions are combined to form seven component scores.<br><br>Each component is scored from 0-3 on a variety of scales.	The final score is the total of each component.<br><br>Original paper isn't clear on how to score some of the components ((e.g., component 3 how to categorise "6"). The measure will be scored in line with this reference: https://biolincc.nhlbi.nih.gov/media/search?q=PSQI)	7 subscales (components): Subjective sleep quality, sleep latency, sleep duration, habitual sleep efficiency, sleep disturbances, use of sleep medication and daytime dysfunction.	None.	None.	A higher score indicates severe difficulties in all areas.
**DePaul symptom questionnaire – post-exertional malaise (DSQ-PEM)**<br>**Ref**: cotler, j., holtzman, c., dudun, c., & jason, l. A. (2018). A brief questionnaire to assess post-exertional malaise. Diagnostics, 8(3), 66.<br>**Scoring**: Jason, Leonard. (2018). DePaul Symptom Questionnaire - Post-Exertional Malaise short form (DSQ-PEM). **HTTPS://WWW.RESEARCHGATE.NET/PUBLICATION/358281945_DEPAUL_SYMPTOM_QUESTIONNAIRE_-_POST-EXERTIONAL_MALAISE_SHORT_FORM_DSQ-PEM**<br>**HTTPS://WWW.COMMONDATAELEMENTS.NINDS.NIH.GOV/SITES/NINDSCDE/FILES/DOC/MECFS/F2771_GUIDANCE_FOR_CORE_PEM_ASSESSMENT.PDF**						

A 10-item questionnaire to assess Post-Exertional Malaise (Pem)	Questions 1 – 5 are rated for frequency and severity: On a 5 point likert scale (0-5).<br><br>Questions 6-8 and 10 are rated Yes/No.<br><br>Question 9 rated 6 point scale.	**DSQ PEM SUUBSCALE:** A frequency of at least 2 and a severity of at least 2 on any one of the 5 questions indicate that PEM is present.<br><br>If threshold is met the DSQ PEM is "Yes;" otherwise it is "No."<br><br>A frequency of 2 on one question and a severity of 2 on a separate question does not satisfy the threshold.	2 – Post-Exertional Malaise scale of the DSQ (Q1-5) and indicator questions (Q6-10)	If all items are missing then Pem not calculated , if 1 item missing then PEM can still be calculated .<br><br>If 1 item in a bunch missing then PEM shouldn't be calculated .		Score indicates if PEM is present or not
**BGSI – GOAL ATTAINMENT AND GOAL SATISFACTION**<br>Clare l, kudlicka a, oyebode jr, et al. Individual goal-oriented cognitive rehabilitation to improve everyday functioning for people with early-stage dementia: a multicentre randomised controlled trial (the great trial). Int j geriatr psychiatry. 2019; 34: 709–721. Https://doi.org/10.1002/gps.5076						
BANGOR GOAL SETTING INTERVIEW	Each scored on a scale from 1 – 10.<br><br>Variables in dataset:<br>Attainment<br>Goal %<br>Satisfaction<br>Generalisation	mean attainment score will be calculated.<br><br>• Mean Attainment Score = Sum of attainment scores (goal 1, goal 2, goal 3) / number of goals set (3)<br><br>Scores range from 0 – 10.	N/A	N/A	N/A	Higher scores indicate better goal attainment and satisfaction

	Readiness<br>Importance<br>difficulty	Using mean scores calculated, attainment change scores can also be calculated:<br>• Change 1 = T1 mean score – Baseline mean score<br>• Change 2 = T2 mean score – Baseline mean score<br>• Change 3 = T2 mean score – T1 mean score<br><br>Goal satisfaction scores will be calculated:<br>• Mean Satisfaction score = Sum of attainment scores (goal 1, goal 2, goal 3) / number of goals set (3)<br><br>Scores range from 0 – 10.				
