# Supplementary material for: Cognitive Rehabilitation and Functional Outcomes in Long COVID–Related Cognitive Impairment: A Randomized Clinical Trial
Source: JAMA Netw Open. 2026 Jul 1;9(7):e2620687. doi: 10.1001/jamanetworkopen.2026.20687 (PMC13324862; doi:10.1001/jamanetworkopen.2026.20687)
Supplement: Supplement 2. — eTable 1. Patient and public involvement and engagement (PPIE) in the CICERO randomized clinical trial eTable 2. Results of regression models run as a sensitivity analysis on a complete case dataset eTable 3. Levels of missing data per measure eTable 4. Results from regression analysis including readiness, difficulty, and importance eTable 5. Cognitive rehabilitation strategies used in the CICERO randomized clinical trial eAppendix. Adapted version of the Bangor Goal-Setting Interview (BGSI) [file jamanetwopen-e2620687-s002.pdf]

Supplemental Online Content

Vanova M, Patel AR, Scott I, et al. Cognitive rehabilitation and functional outcomes in long COVID–related cognitive impairment: a ranzdomized clinical trial. *JAMA Netw Open*. 2026;9(7):e2620687. doi:10.1001/jamanetworkopen.2026.20687

eTable 1. Patient and public involvement and engagement (PPIE) in the CICERO randomized clinical trial

eTable 2. Results of regression models run as a sensitivity analysis on a complete case dataset

eTable 3. Levels of missing data per measure

eTable 4. Results from regression analysis including readiness, difficulty, and importance

eTable 5. Cognitive rehabilitation strategies used in the CICERO randomized clinical trial

eAppendix. Adapted version of the Bangor Goal-Setting Interview (BGSI)

This supplemental material has been provided by the authors to give readers additional information about their work.

© 2026 Vanova M et al. *JAMA Network Open*.

eTable 1. Patient and Public Involvement and Engagement (PPIE) in the CICERO Randomised Controlled Trial

Section and topic	Item
**1. Aims** of PPIE in the study	The aims for PPIE in CICERO were:<br>• To help shape the neuropsychological rehabilitation intervention (developed and tested in the RCT) and how this was to be administered,<br>• To shape the dissemination of outcomes,<br>• To ensure that the research is transparent and relevant to patients, and<br>• To offer insights into the interpretation of emerging findings.
**2. Methods** used for PPIE in the study	Experts by Experience (EbE) group comprised of:<br>• Three individuals living with cognitive impairments associated with Long Covid<br>• Dr Jason Lim (PPI Co-Lead and Co-I, also living with cognitive impairments associated with Long Covid)<br>• Dr Aida Suarez Gonzalez (PPI Co-Lead and Principal Investigator)<br>• Dr Martina Vanova (Research Fellow leading the CICERO cognitive rehabilitation programme).<br>The EbE group worked via remote videoconferencing. Four meetings took place between June and December 2022 during the preparation for the RCT. A subsequent meeting took place in June 2024 to help interpret the initial RCT data.
**3. Results** of PPIE in the study	Dr Lim contributed to the early stages of the development of the funding proposal and research design.<br>The first and second EbE group meetings provided insights into the lived experience of cognitive impairments in long Covid, including:<br>• How executive function impairments affect initiating action and planning<br>• How social and physical environments influence the difficulties associated with cognitive impairments<br>• How cognitive rehabilitation (CR) strategies may interact with non-cognitive long Covid symptoms<br>• How to prevent post-exertional malaise during the CR<br>• Ways of transferring intervention techniques from one situation to others<br>In the third and fourth EbE meeting, the group:<br>• Helped to pilot the goal-setting interview process<br>• Participated in mock CR sessions, which helped the research team design the intervention<br>• Gave insight on the effectiveness of various cognitive strategies and apps in the rehabilitation process<br>• Gave insight into how to maintain participants' engagement and motivation during CR (maintaining a daily log)<br>The fifth EbE group meeting involved a pilot for the focus group and workshop to be run with control participants.

© 2026 Vanova M et al. *JAMA Network Open*.

**4. Discussion**: outcomes of PPIE in the study	The EbE group worked with the research team to provide understandings of the impact of cognitive impairments on everyday life, and insights crucial for the development of the CR. Moreover, the group highlighted the impact of the cognitive impairments on domestic, family, and employment contexts, work-life balance, and various physical environments. This contributed towards the ecological validity of the CR. Moreover, the group provided the wider context of long Covid and the consequent need to validate existing neuropsychological knowledge about cognitive impairments and CR against lived experiences.
**5. Reflections**: critical commentary on PPIE in the study	Recruitment of EbE group members was challenging for multiple reasons, including reluctance due to the effects of the illness. Illness effects also impacted on the ability of EbE group members to regularly participate on the meetings. Nonetheless, the small size of the EbE group was ultimately assessed to be helpful to enable members to maintain attention and engagement despite the impact of fatigue and cognitive impairment. The timetable of EbE group meetings was disrupted by the delayed start of the RCT. This resulted in a longer gap between the fourth (December 2022) and the fifth meeting (June 2024). As a result, not all of the initial EbE group members were able to attend the final meeting. The initial plan for the EbE group to provide insights on the emerging results was not executed. However, the EbE group helped shape the dissemination workshop on CR for the control group. The lessons learned from this challenge can inform incorporating time delays when planning PPIE working processes.

Note: CR – Cognitive Rehabilitation; EbE – Experts by Experience; PPIE – Public Patient Involvement and Engagement; RCT – Randomised Controlled Trial

© 2026 Vanova M et al. *JAMA Network Open*.

eTable 2. The results of the regression models run as a sensitivity analysis on a complete case dataset.

Outcome	Sensitivity analysis	Time point	N	Maximum Likelihood estimates				Adjusted values (M, SE)	
				COEF	SE	p-value	95% CI	TAU	CR
Goal attainment (BGSI)	SA1	3 months	70	2.73	0.54	<0.001**	(1.66 to 3.79)	4.99 (0.47)	7.72 (0.07)
	SA2	3 months	27	3.40	0.13	<0.001**	(3.14 to 3.65)	4.96 (0.90)	8.36 (0.77)
	SA3	3 months	62	2.88	0.30	<0.001**	(2.28 to 3.47)	5.11 (0.45)	7.99 (0.15)
	SA1	6 months	57	1.70	0.45	<0.001**	(0.81 to 2.58)	5.87 (0.30)	7.56 (0.32)
	SA2	6 months	33	1.29	0.58	0.03*	(0.14 to 2.43)	5.86 (0.40)	7.15 (0.41)
Goal satisfaction (BGSI)	SA1	3 months	70	2.74	0.62	<0.001**	(1.53 to 3.95)	4.70 (0.46)	7.44 (0.15)
	SA1	6 months	57	1.40	0.32	<0.001**	(0.77 to 2.04)	5.76 (0.12)	7.16 (0.21)
DKEFS trail making C4 number-letter sequencing	SA1	3 months	61	1.22	0.29	<0.001**	(0.65 to 1.80)	9.17 (0.40)	10.39 (0.11)

Note. BGSI – Bangor Goal-Setting Interview; COEF – coefficient; CR – Cognitive rehabilitation, DKEFS – Delis-Kaplan Executive Function System; SA – Sensitivity analysis; TAU – Treatment as usual. * p value <0.05, ** p values < 0.01.

SA4 – Incorporation of the number of intervention sessions attended. The majority of the sample (N 32) received all 10 CR sessions, some received 1-4 sessions (N 4), and some withdrawn from the CR (N 2). Therefore, this sensitivity analysis would have been uninformative and was not undertaken.
SA5 – Excluding participants where protocol deviations were identified if this rose above 10%. As only three were identified (ineligible participant randomised – N 1, participants received their BGSI goals via email at baseline – N 2), this was not conducted.

© 2026 Vanova M et al. *JAMA Network Open*.

eTable 3: Levels of missing data per measure.

Data	Measure	Variable	N Randomised	Baseline		3 Months		6 Months	
				Obs	Missing (%)	Obs	Missing (%)	Obs	Missing (%)
**Demographic Data**	**Age**	**Overall**	**N = 78**	**78**	**0 (0)**	**-**	**-**	**-**	**-**
		TAU	N = 40	40	0 (0)	-	-	-	-
		CR	N = 38	38	0 (0)	-	-	-	-
	**Site, Sex, Ethnicity, Education (Years), Occupation**	**Overall**	**N = 78**	**78**	**0 (0)**	**-**	**-**	**-**	**-**
		TAU	N = 40	40	0 (0)	-	-	-	-
		CR	N = 38	38	0 (0)	-	-	-	-
	**Disability (Yes/No)**	**Overall**	**N = 78**	**77**	**1 (1.3%)**	**-**	**-**	**-**	**-**
		TAU	N = 40	40	0 (0)	-	-	-	-
		CR	N = 38	37	1 (2.6%)	-	-	-	-
	**Drugs**	**Overall**	**N = 78**	**77**	**1 (1.3%)**	**-**	**-**	**-**	**-**
		TAU	N = 40	**39**	1 (2.5%)	-	-	-	-
		CR	N = 38	38	0 (0)	-	-	-	-
	**CV19 Conditions Prior (Yes/No)**	**Overall**	**N = 78**	**76**	**2 (2.6%%)**	**-**	**-**	**-**	**-**
		TAU	N = 40	40	0 (0)	-	-	-	-
		CR	N = 38	36	2 (5.3%)	-	-	-	-
	**CV19 Conditions During (Yes/No)**	**Overall**	**N = 78**	**77**	**1 (1.3%)**	**-**	**-**	**-**	**-**
		TAU	N = 40	40	0 (0)	-	-	-	-
		CR	N = 38	**37**	1 (2.6%)	-	-	-	-
**Self-Report Outcome Measures**	**BGSI Goal Attainment & Satisfaction & BGSI covariates (Readiness, Difficulty, Importance and Acceptable achievement)**	**Overall**	**N = 78**	**78**	**0 (0)**	**72**	**6 (7.7%)**	**58**	**20 (25.6%)**
		TAU	N = 40	40	0 (0)	39	1 (2.5%)	30	10 (25.0%)
		CR	N = 38	**38**	0 (0)	33	5 (13.2%)	28	10 (26.3%)
	**Life Space Assessment (LSQ)**	**Overall**	**N = 78**	**78**	**0 (0)**	**67**	**11 (14.1%)**	**53**	**23 (29.5%)**
		TAU	N = 40	40	0 (0)	37	3 (7.5%)	29	11 (27.5%)
		CR	N = 38	38	0 (0)	30	8 (21.1%)	26	12 (31.6%)
	**Social Functioning (SF-DEM)**	**Overall**	**N = 78**	**78**	**0 (0)**	**67**	**11 (14.1%)**	**53**	**23 (29.5%)**

© 2026 Vanova M et al. *JAMA Network Open*.

		TAU	N = 40	40	0 (0)	37	3 (7.5%)	29	11 (27.5%)
		CR	N = 38	38	0 (0)	30	8 (21.1%)	26	12 (31.6%)
	**Instrumental Activities Of Daily Living (IADL) Scale**	**Overall**	**N = 78**	**78**	**0 (0)**	**67**	**11 (14.1%)**	**53**	**23 (29.5%)**
		TAU	N = 40	40	0 (0)	37	3 (7.5%)	29	11 (27.5%)
		CR	N = 38	38	0 (0)	30	8 (21.1%)	26	12 (31.6%)
	**Generalised Anxiety Disorder (GAD-7)**	**Overall**	**N = 78**	**78**	**0 (0)**	**67**	**11 (14.1%)**	**53**	**23 (29.5%)**
		TAU	N = 40	40	0 (0)	37	3 (7.5%)	29	11 (27.5%)
		CR	N = 38	38	0 (0)	30	8 (21.1%)	26	12 (31.6%)
	**Patient Health Questionnaire (PHQ-8)**	**Overall**	**N = 78**	**78**	**0 (0)**	**67**	**11 (14.1%)**	**53**	**23 (29.5%)**
		TAU	N = 40	40	0 (0)	37	3 (7.5%)	29	11 (27.5%)
		CR	N = 38	38	0 (0)	30	8 (21.1%)	26	12 (31.6%)
	**Chalder Fatigue Scale (CFS)**	**Overall**	**N = 78**	**78**	**0 (0)**	**67**	**11 (14.1%)**	**53**	**23 (29.5%)**
		TAU	N = 40	40	0 (0)	37	3 (7.5%)	29	11 (27.5%)
		CR	N = 38	38	0 (0)	30	8 (21.1%)	26	12 (31.6%)
	**Pittsburgh Sleep Quality (PSQI)**	**Overall**	**N = 78**	**78**	**0 (0)**	**67**	**11 (14.1%)**	**53**	**23 (29.5%)**
		TAU	N = 40	40	0 (0)	37	3 (7.5%)	29	11 (27.5%)
		CR	N = 38	38	0 (0)	30	8 (21.1%)	26	12 (31.6%)
	**DePaul Symptom – Post-Exertional Malaise (DSQ-PEM)**	**Overall**	**N = 78**	**78**	**0 (0)**	**67**	**11 (14.1%)**	**53**	**23 (29.5%)**
		TAU	N = 40	40	0 (0)	37	3 (7.5%)	29	11 (27.5%)
		CR	N = 38	38	0 (0)	30	8 (21.1%)	26	12 (31.6%)
**Behavioural Data (Cognitive Tests)**	**RBANS**	**Overall**	**N = 78**	**76**	**2 (2.6%)**	**63**	**15 (19.2%)**	**44**	**34 (43.6%)**
		TAU	N = 40	39	1 (2.5%)	36	4 (10.0%)	24	16 (40.0%)
		CR	N = 38	37	1 (2.6%)	27	11 (29.0%)	20	18 (47.4%)
	**DKEFS Trail Making, Outcomes and Errors**	**Overall**	**N = 78**	**76**	**2 (2.6%)**	**63**	**15 (19.2%)**	**44**	**34 (43.6%)**
		TAU	N = 40	39	1 (2.5%)	36	4 (10.0%)	24	16 (40.0%)
		CR	N = 38	37	1 (2.6%)	27	11 (29.0%)	20	18 (47.4%)
	**DKEFS Colour Word, Outcomes**	**Overall**	**N = 78**	**71**	**7 (9.0%)**	**62**	**16 (20.5%)**	**44**	**34 (43.6%)**
		TAU	N = 40	38	2 (5.0%)	36	4 (10.0%)	24	16 (40.0%)
		CR	N = 38	33	5 (13.2%)	26	12 (31.6%)	20	18 (47.4%)
	**DKEFS Colour Word, Errors**	**Overall**	**N = 78**	**75**	**3 (3.9%)**	**63**	**15 (19.2%)**	**44**	**34 (43.6%)**
		TAU	N = 40	39	1 (2.5%)	36	4 (10.0%)	24	16 (40.0%)
		CR	N = 38	36	2 (5.3%)	27	11 (29.0%)	20	18 (47.4%)

© 2026 Vanova M et al. *JAMA Network Open*.

**DKEFS – Verbal Fluency, Outcomes**	**Overall**	**N = 78**	**76**	**2 (2.6%)**	**63**	**15 (19.2%)**	**44**	**34 (43.6%)**
	TAU	N = 40	39	1 (2.5%)	36	4 (10.0%)	24	16 (40.0%)
	CR	N = 38	37	1 (2.6%)	27	11 (29.0%)	20	18 (47.4%)
**WAIS**	**Overall**	**N = 78**	**76**	**2 (2.6%)**	**63**	**15 (19.2%)**	**44**	**34 (43.6%)**
	TAU	N = 40	39	1 (2.5%)	36	4 (10.0%)	24	16 (40.0%)
	CR	N = 38	37	1 (2.6%)	27	11 (29.0%)	20	18 (47.4%)
**Gorilla Data, Outcomes and Errors**	**Overall**	**N = 78**	**74**	**4 (5.1%)**	**62**	**16 (20.5%)**	**44**	**34 (43.6%)**
	TAU	N = 40	37	3 (7.5%)	36	4 (10.0%)	34	16 (40.0%)
	CR	N = 38	37	1 (2.6%)	26	12 (31.6%)	20	18 (47.4%)
**4 mountains, Outcome and Errors**	**Overall**	**N = 78**	**76**	**2 (2.6%)**	**63**	**15 (19.2%)**	**45**	**33 (42.3%)**
	TAU	N = 40	39	1 (2.5%)	36	4 (10.0%)	24	16 (40.0%)
	CR	N = 38	37	1 (2.6%)	27	11 (29.0%)	21	17 (44.7%)

Note. Obs – Observed; TAU – Treatment as Usual arm (i.e. Standard Care); CR – Cognitive Rehabilitation

© 2026 Vanova M et al. *JAMA Network Open*.

eTable 4: Results from regression analysis including Readiness, Difficulty, and Importance.

TIME POINT	*FACTOR*	*MAXIMUM LIKELIHOOD ESTIMATES*						
		COEF (OR)	*SE*	*T*	*P-VALUE*	*95% CI LOWER*	*UPPER*	*SIG*
3 MONTHS	*Group Allocation*	*2.624*	*.471*	*5.57*	*<0.001*	*1.70*	*3.55*	***
	BGSI Readiness	*0.25*	*0.03*	*8.45*	*<0.001*	*0.19*	*0.31*	***
	BGSI Difficulty	*0.14*	*0.03*	*4.61*	*<0.001*	*0.08*	*1.00*	***
	BGSI Importance	*-1.46*	*0.12*	*-1.22*	*0.22*	*-0.38*	*0.09*	
	Goal Acceptance score	*0.02*	*0.23*	*0.09*	*0.93*	*-0.42*	*0.46*	
6 MONTHS	*Group Allocation*	*1.62*	*0.11*	*15.04*	*<0.001*	*1.41*	*1.83*	***
	BGSI Readiness	*0.22*	*0.16*	*1.42*	*0.16*	*-0.09*	*0.53*	
	BGSI Difficulty	*0.07*	*0.16*	*0.45*	*0.67*	*-0.24*	*0.39*	
	BGSI Importance	*-0.08*	*0.17*	*-0.48*	*0.63*	*-0.42*	*0.23*	
	Goal Acceptance score	*-0.53*	*0.09*	*-5.72*	*<0.001*	*-0.72*	*-0.35*	***

Note. Sig - significance; ** p value <0.05, *** p values < 0.01 MI; multiple imputation, CC; Complete Case; TAU – Treatment as Usual arm (i.e. Standard Care); CR – Cognitive Rehabilitation
All results are Allocation: Group 2 (CR) vs Group 1 (TAU)

© 2026 Vanova M et al. *JAMA Network Open*.

eTable 5. Cognitive rehabilitation strategies used in the CICERO Randomised Controlled Trial.

**Cognitive domain**	**Observable problem – behaviour**	**Typical test-based deficit***	**Cognitive rehabilitation strategy**	**Mechanism of action**	**Example from practice**
**Working memory/ Learning**	Difficulty retaining complex information<br><br>Memory complaints<br><br>Memory overload, difficulty remembering (both objective and self-perceived)	Poor encoding and retention on memory tests (e.g., *Hopkins Verbal Learning Test, Rivermead Behavioural Memory Test*); low scores on delayed recall and recognition trials; reduced span on working memory tasks (e.g., *Digit Span, Spatial Span*)	Visualisation	Promotes retention by transforming verbal information into visual imagery anchored in familiar contexts, enhancing encoding and chunking.	*Visualise the events in your calendar for the day with the details, places, etc.*
			Chunking	Facilitates encoding and reduces memory load by restructuring information into manageable units (“chunks”).	*Break the long text into smaller sections. After each paragraph, stop and summarise the main idea in your own words.*
			Structuring techniques	Reduces executive and memory demands by imposing external structure on tasks, promoting more efficient cognitive processing.	*Structure complex information (e.g., work report or document), identify main topics and questions to answer for each point.*
			Categorisation	Improves recall by promoting semantic structuring, which facilitates deeper encoding and increases mnemonic efficiency.	*To remember your shopping list, group the items into categories, e.g., bakery, meat, diary, etc.*
**Sustained attention**	Mind wandering; losing train of thought); difficulty sustaining attention	Reduced vigilance and increased omission errors on continuous	Pacing	Self-pacing supports sustained attention by introducing a control element (structured breaks)[1]. As a self-	*Set a timer for 10 minutes of work followed by a 5-minute breathing break.*

© 2026 Vanova M et al. *JAMA Network Open*.

Cognitive domain	Observable problem – behaviour	Typical test-based deficit*	Cognitive rehabilitation strategy	Mechanism of action	Example from practice
	during reading or having conversations	performance tasks (e.g., *Conners' CPT, TEA – Elevator Counting)*; fluctuating reaction times or rapid performance decay in time-limited tasks (e.g., *PASAT*)		regulatory strategy, it increases perceived control and promotes task persistence [2].	
**Focused attention**	Easily distracted by environmental stimuli (e.g. noises)<br><br>Forgetting ongoing tasks or intentions	Increased distractibility and interference effects on inhibition tasks (e.g. *Stroop Test*); reduced accuracy on simple attention measures such as *TMT Part A* or *TEA Visual Elevator*.	Environment restructuring / Removing distractors	Reduces attentional and memory load by modifying the environment to minimise distractions and support task focus[3].	*Do your activities (e.g., reading, email writing, calendar organising) in a quiet, non-distracting environment. Turn-off phone notifications, ask people not to disturb, etc.*
			Behavioural registry/ Identify distractors	Enhances self-awareness by systematically tracking behaviours, identifying patterns, triggers, and distractors that inform targeted strategies.	*Keep a simple log when you forget something or become distracted. Write down what you forgot, where you were, and what was happening around you. This helps to spot patterns.*

© 2026 Vanova M et al. *JAMA Network Open*.

**Cognitive domain**	**Observable problem – behaviour**	**Typical test-based deficit***	**Cognitive rehabilitation strategy**	**Mechanism of action**	**Example from practice**
**Executive functioning**	Difficulties initiating actions and tasks<br><br>Difficulties to plan or sequence tasks; difficulty generating sub-goals<br><br>Difficulty adapting to novelty or unexpected events	Impaired task-switching and set-shifting (e.g., *D-KEFS TMT);* reduced planning efficiency and rule violations on structured problem-solving tasks (e.g. *Tower of London*, *D-KEFS Tower*); impaired verbal fluency or switching on fluency tasks.	Self-instructions	Directs attention and promotes behavioural self-regulation by internalised language [4].	*Talk yourself through the activity (e.g., cooking) step-by-step, saying what you are going to do.*
			Goal Management Training (Stop & Think)	Promotes executive control by training individuals to pause and reflect before acting, thereby reducing impulsivity and supporting task structuring [5].	*Reflect on your intentions. Before starting the activity, say: "STOP — what's my goal?" For example: "Write the email to confirm dates." List the steps briefly. While writing, pause: "Am I still doing that?" At the end, check: "Did I do what I meant to?"*
			Habit formation (Building routines)	Reduces executive load by establishing predictable, structured activity patterns () [6].	*Do the activity (e.g., reading) at the same time each day so you link it with a specific time and context.*
			External cueing/prompting	Compensates for deficits in initiation, planning, and prospective memory by using externalised prompts (e.g. diaries, calendars, diaries) to cue actions, structure daily routines, gradually promoting internalisation of self-management strategies.	*Set up a calendar notification before each appointment depending on how much time you need to prepare for or drive to the appointment.*

© 2026 Vanova M et al. *JAMA Network Open*.

Cognitive domain	Observable problem – behaviour	Typical test-based deficit*	Cognitive rehabilitation strategy	Mechanism of action	Example from practice
			Visual aids	Provides external cues to guide task initiation and sequencing. Reinforces goal-directed behaviour by prompting attention to sub-tasks and facilitating step-by-step execution.	*Write down a prompt and place it visibly to remind yourself to ask a question during your next call. Use sticky notes or visual checklists to stay on track with the task.*

Note: ADL – Activities of Daily Living; CPT – Continuous Performance Test; IADL – Instrumental ADL; D-KEFS – Delis-Kaplan Executive Function System; PASAT – Paced Auditory Serial Addition Test; TEA – Test of Everyday Attention; TMT – Trial Making Test; VLT – Verbal Learning Test; WM – Working Memory. * Standardised tests routinely used in clinical practice may fail to detect objective cognitive deficits in people with PASC. This may occur despite persistent subjective complaints and observable difficulties in daily tasks.

References

1 Scerbo MW, Greenwald CQ, Sawin DA. The Effects of Subject-Controlled Pacing and Task Type on Sustained Attention and Subjective Workload. *J Gen Psychol* 1993; **120**: 293–307.

2 Sohlberg MM, Mateer CA. Improving Attention and Managing Attentional Problems. *Ann N Y Acad Sci* 2006; **931**: 359–75.

3 Wilson BA. Memory rehabilitation. *Neuropsychology of Memory* 2003; : 263.

4 Wood RLl, Worthington AD. Neurobehavioural rehabilitation: A conceptual paradigm. In: Neurobehavioural Disability and Social Handicap Following Traumatic Brain Injury, 1st edition. Psychology Press, 2001.

5 Gillen G. Managing Executive Function Impairments to Optimize Function. In: Cognitive and Perceptual Rehabilitation. Elsevier, 2009: 245–83.

6 Evans JJ, Emslie H, Wilson BA. External cueing systems in the rehabilitation of executive impairments of action. *Journal of the International Neuropsychological Society* 1998; **4**: 399–408.

© 2026 Vanova M et al. *JAMA Network Open*.

eAppendix. Adapted version of the Bangor Goal-Setting Interview (BGSI)

Bangor Goal-Setting Interview (BGSI) questions.

CICERO - BGSI online

Thank you for taking part in our study. In the next few minutes, we will define three personal goals that you would like to achieve if allocated to the intervention group of this study. This process will take approximately 30 minutes, and I will be taking notes during this process.

We determine the three goals before each participant is randomly allocated to either the control or the intervention group. I would like to emphasise that this process is completely independent of us as researchers or your (participant's) responses.

Do you have any questions before we start?

Areas of Concern

Q1. *Please, tell me briefly about your post-infection recovery.*

- a) *Has your cognition spontaneously improved over time?*
- b) *Have you undergone any cognitive rehabilitation before?*
- c) *Do you feel that your cognition is currently improving, worsening, or is it stable?*

Q2. *Have you received any information or training on fatigue management?*
(Specify what information was received and when. If not, why not?)

- Yes (Specify when and how)
- No

Q3. *To begin with, we need to identify the areas where you may have some problems or limitations.* (Ask each question separately and give examples.)

- *Is there something you have stopped doing because of your cognitive difficulties, and would you like to start doing it again? For instance, reading newspapers, shopping, etc.* (Please specify):
- *Are there things you would like to manage better? For instance, day-to-day activities planning, etc.* (Please specify):

Setting Goal 1 [1]

(The following questions are also done for GOALS 2 and 3. The areas of concern specified above will inform the following process. Each goal is SMART: specific, measurable, attainable, reasonable, and time-bound. Each goal follows this structure: I will (action) (object of the action) (how) (time frame or frequency).

Q4. *What is one of the three most important activities (Q3) you would like to work on first?*

Q5. (Identify the cognitive domains related to GOAL 1 that the participant would like to work on to improve. Check with the participant if they have problems with any of the following by giving examples of problems for each domain.)

- Problems remembering new information, difficulties following instructions or completing several-step tasks, losing one's own 'train of thought' (Working memory)

[1] Repeat for Goals 2 and 3

© 2026 Vanova M et al. *JAMA Network Open*.

- Mind wandering after a short time, being easily distracted, problems keeping focus on a task (Attention)
- Problems initiating tasks, planning and executing complex tasks, and/or dealing with novelty (Executive functioning & Planning)
- Other (Please specify):

Q6. (Goal structure: I will (action) (object of the action) (how) (time frame or frequency). Select the "action"):
- remember
- expand
- focus on
- read
- initiate
- finish
- execute
- plan
- create
- overcome
- Other (Please specify):

Q7. (Goal structure: I will (Q6) (object of the action) (how) (time frame or frequency. Please specify the "object of action"):

Q8. Goal structure: I will (Q6) (Q7) (how) (time frame or frequency).

(Choose from the following or define your own "how"):
- alone, without help
- without interruption
- other (Please specify):

Q9. Goal structure: I will (Q6) (Q7) (Q8) (time frame or frequency).
(Choose from the following "time frame or frequency"):
- Timeframe: E.g., in 2 (days, weeks, months), by May 2023, etc. (Please specify):
- Frequency: E.g., 5 times a day, week, month, every time, etc. (Please specify):

Q10. Goal 1: I will (Q6) (Q7) (Q8) (Q9).
(Here you can rephrase Goal 1. Type the final goal phrase. Check if Goal 1 complies with the following:
Specific: One specific action or challenge per goal. Observable behaviour.
Measurable: Behaviour (goal) can be observed and evaluated by others.
Attainable: You must be able to stick to the goal regularly. Appealing, the patient is motivated to do it regularly. Consider the patient's limitations.
Realistic: The goal has to be within the scope of a cognitive rehabilitation programme.
Time-bound: The goal specifies a time frame or frequency of behaviour.)

Scoring Goal 1 [2]

Q11. Goal 1.

---

[2] Repeat for Goals 2 and 3

© 2026 Vanova M et al. *JAMA Network Open*.

What is your current performance on this goal? How much are you able to do it?"
(Read the goal and enter the current attainment.)

Q12. *On a scale from 1 to 10, how much are you able to do the current goal?*

Q13. *On a scale from 1 to 10, how SATISFIED are you with your current Goal performance?*

Q14. *If you were not able to achieve the goal fully, what would be an acceptable achievement?*
(Read the goal and enter the 50% attainment.)

Q15. *On a scale from 1 to 10, how much would you score the acceptable (50%) achievement?*

Q16. *On a scale from 1 to 10, how READY do you feel to make changes to improve on your problem?*

Q17. *On a scale from 1 to 10, how DIFFICULT do you feel it will be to improve on your problem?*

Q18. *On a scale from 1 to 10, how IMPORTANT is this goal for you to achieve?*

© 2026 Vanova M et al. *JAMA Network Open*.
