# Supplementary material for: Cognitive Rehabilitation and Functional Outcomes in Long COVID–Related Cognitive Impairment: A Randomized Clinical Trial
Source: JAMA Netw Open. 2026 Jul 1;9(7):e2620687. doi: 10.1001/jamanetworkopen.2026.20687 (PMC13324862; doi:10.1001/jamanetworkopen.2026.20687)
Supplement: Supplement 3. — Data Sharing Statement [file jamanetwopen-e2620687-s003.pdf]

Data Sharing Statement

Vanova. Cognitive Rehabilitation and Functional Outcomes in Long COVID–Related Cognitive Impairment: A Randomized Clinical Trial. *JAMA Netw Open*. Published online July 1, 2026. doi:10.1001/jamanetworkopen.2026.20687

Data

Additional Information: Trial registration number NCT05731570, ClinicalTrials.gov registry, registered on 9th February 2023. https://clinicaltrials.gov/study/NCT05731570
Data available: Yes
Data types: Deidentified participant data, Data dictionary
How to access data: Data can be made available upon request to: Dr Martina Vanova (m.vanova@ucl.ac.uk)
When available: With publication

Supporting Documents

Document types: Statistical/analytic code, Informed consent form
How to access documents: Data can be made available upon request to: Dr Martina Vanova (m.vanova@ucl.ac.uk)
When available: With publication

Additional Information

Who can access the data: Researchers whose proposed use of the data has been approved
Types of analyses: Legitimate, topic-related analysis.
Mechanisms of data availability: With a signed data access agreement
